# Supplementary figures and images for: Identification of O-glycosylation related genes and subtypes in ulcerative colitis based on machine learning
Source: PLoS One. 2024 Dec 31;19(12):e0311495. doi: 10.1371/journal.pone.0311495 (PMC11687659; doi:10.1371/journal.pone.0311495)

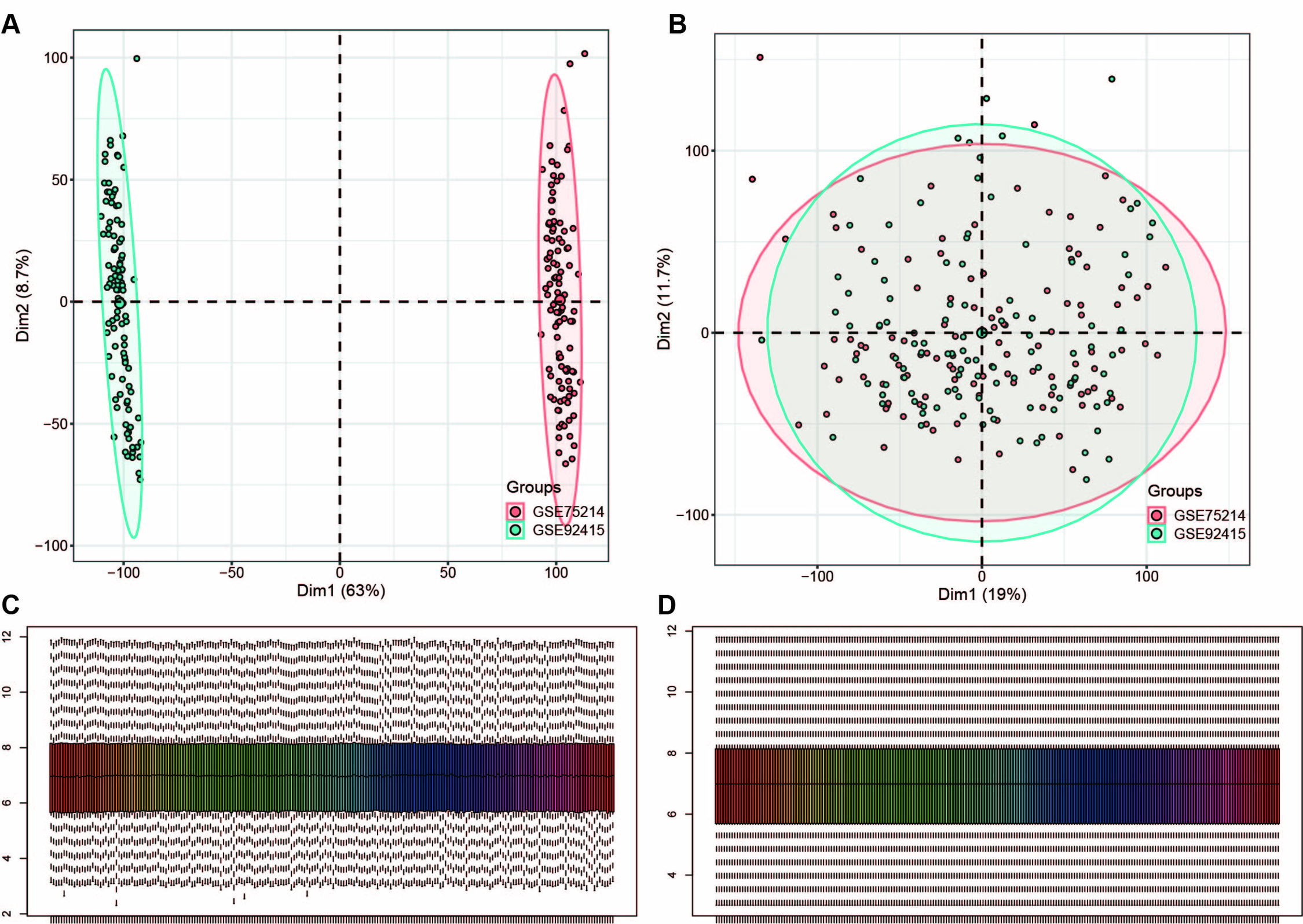

Supplement: S1 Fig — (A-B) GSE75214 and GSE92415 combined and used R packages "limma" and "sva" to remove batch effects, resulting in 16,467 genes and 216 samples. A: before merging; and B: after merging. (C-D) R language preprocessCore package homogenized the dataset; C: before homogenization; and D: after homogenization. (TIF) [file pone.0311495.s001.tif]

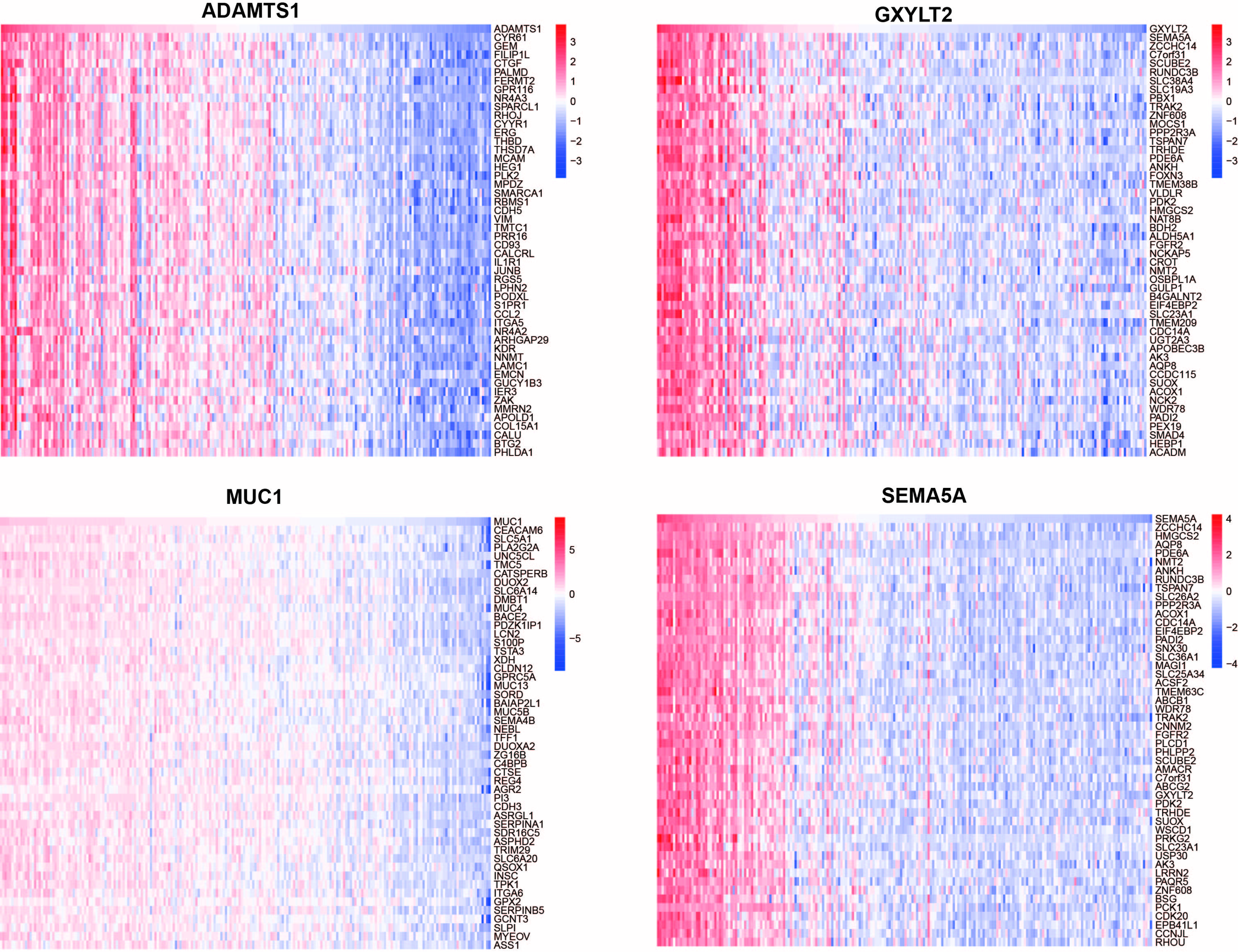

Supplement: S2 Fig — The positive correlation between the top 50 genes and 4 hub genes was displayed using heatmaps. (TIF) [file pone.0311495.s002.tif]
