# Supplementary material for: Identification of O-glycosylation related genes and subtypes in ulcerative colitis based on machine learning
Source: PLoS One. 2024 Dec 31;19(12):e0311495. doi: 10.1371/journal.pone.0311495 (PMC11687659; doi:10.1371/journal.pone.0311495)
Supplement: S2 File — This is the R language code used by the bioinformatics method involved in this study. (DOCX) [file pone.0311495.s004.docx]

1. **Merge and deduplication of datasets**

rm(list = ls())

#引用包

library(limma)

library(sva)

library(tidyverse)

library(limma)

library(ggrepel)

library(ggthemes)

library(tidyverse)

library(pheatmap)

Sys.setenv(LANGUAGE = "en") #显示英文报错信息

options(stringsAsFactors = FALSE) #禁止chr转成factor

#均一化处理

express <- read.csv("GSE75214矩阵.csv", row.names = 1)

group_list <- read.table("GSE75214样本.txt", sep = "\t", header = T)

express<-dplyr::select(express,group_list$Accession)

# 针对复杂基因名的状况

# express$ID=NA

# for (i in 1:nrow(express)) {

# express$ID[i]<-unlist(str_split_fixed(rownames(express)[i],"//",3))[2]

# }

# express$ID<-str_replace_all(express$ID," ","")

# express<-distinct(express,ID,.keep_all = T)

# rownames(express)<-express$ID

# express<-dplyr::select(express,-ID)

#去掉一个探针对应多个基因的结果

express<-express[which(unlist(str_split_fixed(rownames(express),"///",2))[,2]==""),]

express=na.omit(express)

range(express)

# if yes no need to log transfer, if above this range, have to do log transfer.

# express <- log2(express+1)

# range(express)

express=rbind(geneNames=colnames(express), express)

write.table(express, file="GSE75214.txt", sep="\t", quote=F, col.names=F)

rm(list = ls())

files=c("GSE75214.txt", "GSE92415.txt")

#获取交集基因

geneList=list()

for(i in 1:length(files)){

inputFile=files[i]

rt=read.table(inputFile, header=T, sep="\t",check.names=F)

header=unlist(strsplit(inputFile, "\\.|\\-"))

geneList[[header[1]]]=as.vector(rt[,1])

}

intersectGenes=Reduce(intersect, geneList)

#数据合并

allTab=data.frame()

batchType=c()

for(i in 1:length(files)){

inputFile=files[i]

header=unlist(strsplit(inputFile, "\\.|\\-"))

#读取输入文件，并对输入文件进行整理

rt=read.table(inputFile, header=T, sep="\t", check.names=F)

rt=as.matrix(rt)

rownames(rt)=rt[,1]

exp=rt[,2:ncol(rt)]

dimnames=list(rownames(exp),colnames(exp))

data=matrix(as.numeric(as.matrix(exp)),nrow=nrow(exp),dimnames=dimnames)

rt=avereps(data)

colnames(rt)=paste0(header[1], "_", colnames(rt))

#对数值大的数据取log2

qx=as.numeric(quantile(rt, c(0, 0.25, 0.5, 0.75, 0.99, 1.0), na.rm=T))

LogC=( (qx[5]>100) || ( (qx[6]-qx[1])>50 && qx[2]>0) )

if(LogC){

rt[rt<0]=0

rt=log2(rt+1)}

if(header[1] != "TCGA"){

rt=normalizeBetweenArrays(rt)

}

#数据合并

if(i==1){

allTab=rt[intersectGenes,]

}else{

allTab=cbind(allTab, rt[intersectGenes,])

}

batchType=c(batchType, rep(i,ncol(rt)))

}

#对数据进行矫正，输出矫正后的结果

outTab=ComBat(allTab, batchType, par.prior=TRUE)

library(FactoMineR)

library(factoextra)

ddb.pca <- PCA(t(allTab), graph = FALSE)

pheno<-data.frame(ID=colnames(allTab))

pheno$cancer<-pheno$ID

pheno[1:108,2]<-"GSE75214"

pheno[109:216,2]<-"GSE92415"

pdf(file = "去除批次效应前.pdf",height=8,width=8)

fviz_pca_ind(ddb.pca,

geom.ind = "point", # 只显示点

pointsize =2, # 点的大小

pointshape = 21, # 点的形状

fill.ind = pheno$cancer, # 分组颜色

palette = "lacent", # c("#00AFBB", "#E7B800", "#FC4E07")

addEllipses = TRUE, # 增加置信椭圆

legend.title = "Groups", # 图例标题

title="") +

theme_bw() + # 和ggplot2对接进行美化

theme(text=element_text(size=14,face="plain",color="black"),

axis.title=element_text(size=16,face="plain",color="black"),

axis.text = element_text(size=14,face="plain",color="black"),

legend.title = element_text(size=16,face="plain",color="black"),

legend.text = element_text(size=14,face="plain",color="black"),

legend.background = element_blank(),

legend.position=c(0.9,0.1)

)

dev.off()

library(FactoMineR)

library(factoextra)

ddb.pca <- PCA(t(outTab), graph = FALSE)

pdf(file = "去除批次效应后.pdf",height=8,width=8)

fviz_pca_ind(ddb.pca,

geom.ind = "point", # 只显示点

pointsize =2, # 点的大小

pointshape = 21, # 点的形状

fill.ind = pheno$cancer, # 分组颜色

palette = "lacent", # c("#00AFBB", "#E7B800", "#FC4E07")

addEllipses = TRUE, # 增加置信椭圆

legend.title = "Groups", # 图例标题

title="") +

theme_bw() + # 和ggplot2对接进行美化

theme(text=element_text(size=14,face="plain",color="black"),

axis.title=element_text(size=16,face="plain",color="black"),

axis.text = element_text(size=14,face="plain",color="black"),

legend.title = element_text(size=16,face="plain",color="black"),

legend.text = element_text(size=14,face="plain",color="black"),

legend.background = element_blank(),

legend.position=c(0.9,0.1)

)

dev.off()

library(data.table)

clin=fread("clinicaldata.txt",header = T)

clin=dplyr::filter(clin,condition == "UC")

clin=clin$Accession

colnames(outTab)=str_replace_all(colnames(outTab),"GSE75214_","")

colnames(outTab)=str_replace_all(colnames(outTab),"GSE92415_","")

outTab1=outTab[,clin]

outTab1=as.data.frame(outTab1)

outTab=as.data.frame(outTab)

save(outTab,file = "merge_all.RDATA")

outTab=rbind(geneNames=colnames(outTab), outTab)

write.table(outTab, file="merge_all.txt", sep="\t", quote=F, col.names=F)

save(outTab1,file = "merge_UC.RDATA")

outTab1=rbind(geneNames=colnames(outTab1), outTab1)

write.table(outTab1, file="merge_UC.txt", sep="\t", quote=F, col.names=F)

1. **Identification of differential expressed genes**

rm(list = ls())

library(limma)

library(ggrepel)

library(ggthemes)

library(tidyverse)

library(pheatmap)

Sys.setenv(LANGUAGE = "en") #显示英文报错信息

options(stringsAsFactors = FALSE) #禁止chr转成factor

#均一化处理

load("merge_all.RDATA")

express <- outTab

group_list <- read.table("clinicaldata.txt", sep = "\t", header = T)

express<-dplyr::select(express,group_list$Accession)

# 针对复杂基因名的状况

# express$ID=NA

# for (i in 1:nrow(express)) {

# express$ID[i]<-unlist(str_split_fixed(rownames(express)[i],"//",3))[2]

# }

# express$ID<-str_replace_all(express$ID," ","")

# express<-distinct(express,ID,.keep_all = T)

# rownames(express)<-express$ID

# express<-dplyr::select(express,-ID)

#去掉一个探针对应多个基因的结果

express<-express[which(unlist(str_split_fixed(rownames(express),"///",2))[,2]==""),]

express=na.omit(express)

range(express)

# if yes no need to log transfer, if above this range, have to do log transfer.

#express <- log2(express+1)

cols=rainbow(ncol(express)) ###针对24个样本，设置颜色，整体呈现彩虹色

pdf(file="均一化前.PDF", width=10, height=6)

boxplot(express,outline = F,col =cols) #均一化前

dev.off()

# Normalise data quantiles

library(preprocessCore)

express.norm <- normalize.quantiles(as.matrix(express))

colnames(express.norm) <- colnames(express)

rownames(express.norm) <- rownames(express)

cols=rainbow(ncol(express.norm)) ###针对24个样本，设置颜色，整体呈现彩虹色

pdf(file="均一化后.PDF", width=10, height=6)

boxplot(express.norm,outline = F,col =cols) #均一化前

dev.off()

#不需要再做均一化处理，就只运行这一行：

#express.norm <- express

write.csv(express.norm,file = "merge矩阵new.csv",row.names = T)

#差异分析

design <- model.matrix(~ 0 + factor(group_list$condition))

colnames(design) <- levels(factor(group_list$condition))

rownames(design) <- colnames(express.norm)

design

# 构建差异比较矩阵

contrast.matrix <- makeContrasts(UC-Control, levels = design)

# 至此，差异表达矩阵已构建好

fit <- lmFit(express.norm,design)

fit2 <- contrasts.fit(fit,contrast.matrix)

fit2 <- eBayes(fit2)

# 得到两两差异表达的结果

# b vs. a

x <- topTable(fit2, coef = 1, n = Inf, adjust.method = "BH", sort.by = "P")

#把全部基因的limma分析结果保存到文件

write.csv(x, "差异分析结果.csv", quote = F)

p.cut<-0.05

logFC.cut<-1

volcano<-x

volcano$type[(volcano$adj.P.Val > p.cut|volcano$adj.P.Val=="NA")|(volcano$logFC < logFC.cut)& volcano$logFC > -logFC.cut] <- "none significant"

volcano$type[volcano$adj.P.Val <= p.cut & volcano$logFC >= logFC.cut] <- "up-regulated"

volcano$type[volcano$adj.P.Val <= p.cut & volcano$logFC <= -logFC.cut] <- "down-regulated"

p = ggplot(volcano,aes(logFC,-1*log10(adj.P.Val),color=type))

p + geom_point()

x_lim <- max(volcano$logFC,-x$logFC)

gg=p + geom_point( aes(size = abs(logFC)),alpha = 0.4) + xlim(-x_lim,x_lim) +labs(x="log2(FC)",y="-log10(adj.P.Val)")+

scale_color_manual(values =c("blue","grey","red"))+

geom_hline(aes(yintercept=-1*log10(p.cut)),colour="black", linetype="dashed") +

geom_vline(xintercept=c(-logFC.cut,logFC.cut),colour="black", linetype="dashed")

print(gg)

pdf(file="火山图.PDF", width=6, height=6)

gg

dev.off()

#热图，选取高低表达top10

volcano1<-dplyr::filter(volcano,adj.P.Val < 0.05)

volcano1<-volcano1[order(volcano1$logFC,decreasing = T),]

aaa<-length(rownames(volcano1))

genename<-c(rownames(volcano1)[1:20],rownames(volcano1)[(aaa-19):aaa])

express1<-as.data.frame(express.norm)

express1$ID<-rownames(express1)

phonedata<-dplyr::filter(express1,ID %in% genename)

phonedata<-dplyr::select(phonedata,-ID)

group_list<-group_list[order(group_list$condition,decreasing = T),]

group_list[,2]

annotation_col<-data.frame(group_list[,2])

rownames(annotation_col)<-group_list[,1]

colnames(annotation_col)<-"Group"

phonedata<-select(phonedata,rownames(annotation_col))

pheatmap(phonedata,scale = "row",cluster_row=T,cluster_col=F, annotation_col = annotation_col,show_colnames = F

,border_color = F,color = colorRampPalette(c("blue", "white", "red"))(50))

dev.off()

pdf(file="热图.PDF", width=6, height=6)

pheatmap(phonedata,scale = "row",cluster_row=T,cluster_col=F, annotation_col = annotation_col,show_colnames = F

,border_color = F,color = colorRampPalette(c("blue", "white", "red"))(50))

dev.off()

#差异基因交集

#差异基因交集

rm(list = ls())

options(stringsAsFactors = FALSE)

library(tidyverse)

logFC_filter=1

adj.P.Val_filter=0.05

merge<-read.csv("差异分析结果.csv",header = T,sep = ",")

colnames(merge)[1]<-"ID"

merge<-filter(merge,adj.P.Val < adj.P.Val_filter)

dat<-filter(merge,logFC >logFC_filter)

merge_UP<-dat$ID

dat<-filter(merge,logFC < -(logFC_filter))

merge_DOWN<-dat$ID

O_GlcNAcylation=read.table("O-GlcNAcylation.txt",header=T)

O_GlcNAcylation=O_GlcNAcylation[,1]

library(VennDiagram)

library(RColorBrewer)

aaa<-list(merge_UP=merge_UP , O_GlcNAcylation=O_GlcNAcylation )

p = venn.diagram(aaa,fill = c(brewer.pal(7,"Set1")[1:2]),

alpha = c(0.5, 0.5), cex = 2,cat.dist = c(0,0.025),

cat.cex=1.5,lty =2, fontfamily ="sans",fontface = "bold",cat.fontfamily = "sans",cat.fontface = "bold",

resolution =300,filename = NULL)

pdf("上调交集.pdf")

grid.draw(p)

dev.off()

aaa<-list(merge_DOWN=merge_DOWN , O_GlcNAcylation=O_GlcNAcylation)

p = venn.diagram(aaa,fill = c(brewer.pal(7,"Set1")[1:2]),

alpha = c(0.5,0.5), cex = 2,cat.dist = c(0, 0.025),

cat.cex=1.5,lty =2, fontfamily ="sans",fontface = "bold",cat.fontfamily = "sans",cat.fontface = "bold",

resolution =300,filename = NULL)

pdf("下调交集.pdf")

grid.draw(p)

dev.off()

a<-intersect(merge_UP,O_GlcNAcylation)

upgene<-unique(c(a))

d<-intersect(merge_DOWN,O_GlcNAcylation)

downgene<-unique(c(d))

difgene<-c(upgene,downgene)

upgene<-data.frame(genename=upgene)

downgene<-data.frame(genename=downgene)

difgene<-data.frame(genename=difgene)

write.table(difgene,"差异基因.txt",row.names = F,quote = F)

write.table(upgene,"差异上调基因.txt",row.names = F,quote = F)

write.table(downgene,"差异下调基因.txt",row.names = F,quote = F)

difgeneall<-c(merge_UP,merge_DOWN)

write.table(difgeneall,"ALL差异基因.txt",row.names = F,quote = F)

1. **Enrichment analyses**

rm(list=ls())

#基因ID转换

library(data.table)

library(org.Hs.eg.db)

library(clusterProfiler)

library(biomaRt)

library(enrichplot)

library(tidyverse)

genelist_input <- fread(file="差异基因.txt", header = T, sep='\t', data.table = F)

genename <- as.character(genelist_input[,1]) #提取第一列基因名

#x - 基因组注释R包

#keys - 需要转换的基因列表

#keytype - 基因名类型

#columns - 希望返回的基因名类型数据，如返回NCBI基因ID使用 ENTREZID

gene_map <- biomaRt::select(org.Hs.eg.db, keys=genename, keytype="SYMBOL", columns=c("ENTREZID"))

gene_map

write.csv(as.data.frame(gene_map),"基因转换.csv",row.names =F)#导出结果至默认路径下

genelist_input<-gene_map[,2]

head(genelist_input)

genelist_input<-na.omit(genelist_input)

#GO分析

Go_result_BP <- enrichGO(genelist_input, 'org.Hs.eg.db', ont="BP", pvalueCutoff=1) #基因ID类型为ENSEMBL的ID形式，选择BP功能组，以P值0.05为界限

goplot(Go_result_BP, showCategory=5) #GO拓扑图

p1<-dotplot(Go_result_BP, showCategory=20) #气泡图，显示前二十个

p1<-p1 + scale_y_discrete(labels=function(x) str_wrap(x, width=50))

ggsave(p1,filename ='GO_BP气泡图.pdf',width = 7.23,height = 8)

ggsave(p1,filename ='GO_BP气泡图.TIFF',width = 7.23,height = 8)

barplot(Go_result_BP, showCategory=20) #条形图，显示前二十个

y=as.data.frame(Go_result_BP)

y$geneID=as.character(sapply(y$geneID,function(x)paste(gene_map$SYMBOL[match(strsplit(x,"/")[[1]],as.character(gene_map$ENTREZID))],collapse="/")))

write.csv(y,"GO-BP.csv",row.names =F)#导出结果至默认路径下。

save(y,file = 'GO-BP.RDATA')

Go_result_CC <- enrichGO(genelist_input, 'org.Hs.eg.db', ont="CC", pvalueCutoff=10) #基因ID类型为ENSEMBL的ID形式，选择CC功能组，以P值0.05为界限

goplot(Go_result_CC, showCategory=5) #GO拓扑图

p1<-dotplot(Go_result_CC, showCategory=20) #气泡图，显示前二十个

p1<-p1 + scale_y_discrete(labels=function(x) str_wrap(x, width=50))

ggsave(p1,filename ='GO_CC气泡图.pdf',width = 7.23,height = 8)

ggsave(p1,filename ='GO_CC气泡图.TIFF',width = 7.23,height = 8)

barplot(Go_result_CC, showCategory=20) #条形图，显示前二十个

y=as.data.frame(Go_result_CC)

y$geneID=as.character(sapply(y$geneID,function(x)paste(gene_map$SYMBOL[match(strsplit(x,"/")[[1]],as.character(gene_map$ENTREZID))],collapse="/")))

write.csv(y,"GO-CC.csv",row.names =F)#导出结果至默认路径下

save(y,file = 'GO-CC.RDATA')

Go_result_MF <- enrichGO(genelist_input, 'org.Hs.eg.db',ont="MF", pvalueCutoff=1000) #基因ID类型为ENSEMBL的ID形式，选择MF功能组，以P值0.05为界限

goplot(Go_result_MF, showCategory=5) #GO拓扑图

p1<-dotplot(Go_result_MF, showCategory=20) #气泡图，显示前二十个

p1<-p1 + scale_y_discrete(labels=function(x) str_wrap(x, width=50))

ggsave(p1,filename ='GO_MF气泡图.pdf',width = 7.23,height = 8)

ggsave(p1,filename ='GO_MF气泡图.TIFF',width = 7.23,height = 8)

barplot(Go_result_MF, showCategory=20) #条形图，显示前二十个

y=as.data.frame(Go_result_MF)

y$geneID=as.character(sapply(y$geneID,function(x)paste(gene_map$SYMBOL[match(strsplit(x,"/")[[1]],as.character(gene_map$ENTREZID))],collapse="/")))

write.csv(y,"GO-MF.csv",row.names =F)#导出结果至默认路径下

save(y,file = 'GO-MF.RDATA')

## GO分析#BP MF CC一起分析

go <- enrichGO(genelist_input, OrgDb = "org.Hs.eg.db", ont="all")

library(ggplot2)

p <- dotplot(go, split="ONTOLOGY") +facet_grid(ONTOLOGY~., scale="free")

p <-p + scale_y_discrete(labels=function(x) str_wrap(x, width=50))

ggsave(p,filename ='GO三合一气泡图.pdf',width = 7.23,height = 8)

ggsave(p,filename ='GO三合一气泡图.TIFF',width = 7.23,height = 8)

write.csv(as.data.frame(go),"GO.csv",row.names =F)#导出结果至默认路径下

#KEGG分析

KEGG_result <- enrichKEGG(genelist_input, keyType = "kegg",pvalueCutoff=1,qvalueCutoff=1,pAdjustMethod = "BH", minGSSize = 5, maxGSSize = 500,organism = "hsa", use_internal_data=T) #KEGG富集分析

barplot(KEGG_result, showCategory=20)#绘制条形图

p1<-dotplot(KEGG_result, showCategory=20) #气泡图，显示前二十个

p1<-p1 + scale_y_discrete(labels=function(x) str_wrap(x, width=50))

ggsave(p1,filename ='KEGG气泡图.pdf',width = 7.23,height = 8)

ggsave(p1,filename ='KEGG气泡图.TIFF',width = 7.23,height = 8)

#圈图

pdf(file="KEGG_circos.pdf",width = 10,height = 7)

kkx=setReadable(KEGG_result, 'org.Hs.eg.db', 'ENTREZID')

cnetplot(kkx, showCategory = 5, circular = TRUE, colorEdge = TRUE,node_label="all")

dev.off()

x=as.data.frame(KEGG_result)

x$geneID=as.character(sapply(x$geneID,function(x)paste(gene_map$SYMBOL[match(strsplit(x,"/")[[1]],as.character(gene_map$ENTREZID))],collapse="/")))

write.csv(as.data.frame(x),"KEGG.csv",row.names =F)#导出结果至默认路径下

save(x,file = 'KEGG.RDATA')

#用P值来排序，不用padjust

#首先，我获取了富集对象x中的数据框，这是S4对象，用@符号来获取,有293行，是没有筛选过的数据

y =KEGG_result@result

#我们的横坐标有问题，是因为这里的GeneRatio是字符串，我们现在要把它变成一个数值

## 分别后去分号前面和后面的数，并变成数值

forward <- as.numeric(sub("/\\d+$", "", y$GeneRatio))

backward <- as.numeric(sub("^\\d+/", "", y$GeneRatio))

## 增加数值表示的一列GeneRatio

y$GeneRatio = forward/backward

showCategory =20

#再设定一个字体大小，大小后期可以调整

font.size =12

library(ggplot2)

library(forcats)

library(dplyr)

#复现气泡图

AAA=y %>%

## 安装p值排序，选区既定数目的行

arrange(pvalue) %>%

slice(1:showCategory)

## 开始ggplot2 作图，其中fct_reorder调整因子level的顺序

p1=ggplot(AAA,aes(GeneRatio,forcats::fct_reorder(Description,Count)))+

## 画出点图

geom_point(aes(color=pvalue, size = Count)) +

## 调整颜色，guide_colorbar调整色图的方向

scale_color_continuous(low="red", high="blue", guide=guide_colorbar(reverse=TRUE))+

## 调整泡泡的大小

scale_size_continuous(range=c(3, 8))+

## 如果用ylab("")或出现左侧空白

labs(y=NULL) +

## 如果没有这一句，上方会到顶

ggtitle("")+

## 设定主题

theme_bw() +

theme(axis.text.x = element_text(colour = "black",

size = font.size, vjust =1 ),

axis.text.y = element_text(colour = "black",

size = font.size, hjust =1 ),

axis.title = element_text(margin=margin(10, 5, 0, 0),

color = "black",size = font.size),

axis.title.y = element_text(angle=90))

p1<-p1 + scale_y_discrete(labels=function(x) str_wrap(x, width=50))

ggsave(filename ='KEGG气泡图2.pdf',width = 8,height = 8)

ggsave(filename ='KEGG气泡图2.TIFF',width = 8,height = 8)

#用P值来排序，不用padjust

#首先，我获取了富集对象x中的数据框，这是S4对象，用@符号来获取,有293行，是没有筛选过的数据

y =Go_result_MF@result

#我们的横坐标有问题，是因为这里的GeneRatio是字符串，我们现在要把它变成一个数值

## 分别后去分号前面和后面的数，并变成数值

forward <- as.numeric(sub("/\\d+$", "", y$GeneRatio))

backward <- as.numeric(sub("^\\d+/", "", y$GeneRatio))

## 增加数值表示的一列GeneRatio

y$GeneRatio = forward/backward

showCategory =20

#再设定一个字体大小，大小后期可以调整

font.size =12

library(ggplot2)

library(forcats)

library(dplyr)

#复现气泡图

AAA=y %>%

## 安装p值排序，选区既定数目的行

arrange(pvalue) %>%

slice(1:showCategory)

## 开始ggplot2 作图，其中fct_reorder调整因子level的顺序

p1=ggplot(AAA,aes(GeneRatio,forcats::fct_reorder(Description,Count)))+

## 画出点图

geom_point(aes(color=pvalue, size = Count)) +

## 调整颜色，guide_colorbar调整色图的方向

scale_color_continuous(low="red", high="blue", guide=guide_colorbar(reverse=TRUE))+

## 调整泡泡的大小

scale_size_continuous(range=c(3, 8))+

## 如果用ylab("")或出现左侧空白

labs(y=NULL) +

## 如果没有这一句，上方会到顶

ggtitle("")+

## 设定主题

theme_bw() +

theme(axis.text.x = element_text(colour = "black",

size = font.size, vjust =1 ),

axis.text.y = element_text(colour = "black",

size = font.size, hjust =1 ),

axis.title = element_text(margin=margin(10, 5, 0, 0),

color = "black",size = font.size),

axis.title.y = element_text(angle=90))

p1<-p1 + scale_y_discrete(labels=function(x) str_wrap(x, width=50))

ggsave(filename ='Go_result_MF2.pdf',width = 8,height = 8)

ggsave(filename ='Go_result_MF2.TIFF',width = 8,height = 8)

#用P值来排序，不用padjust

#首先，我获取了富集对象x中的数据框，这是S4对象，用@符号来获取,有293行，是没有筛选过的数据

y =Go_result_BP@result

#我们的横坐标有问题，是因为这里的GeneRatio是字符串，我们现在要把它变成一个数值

## 分别后去分号前面和后面的数，并变成数值

forward <- as.numeric(sub("/\\d+$", "", y$GeneRatio))

backward <- as.numeric(sub("^\\d+/", "", y$GeneRatio))

## 增加数值表示的一列GeneRatio

y$GeneRatio = forward/backward

showCategory =20

#再设定一个字体大小，大小后期可以调整

font.size =12

library(ggplot2)

library(forcats)

library(dplyr)

#复现气泡图

AAA=y %>%

## 安装p值排序，选区既定数目的行

arrange(pvalue) %>%

slice(1:showCategory)

## 开始ggplot2 作图，其中fct_reorder调整因子level的顺序

p1=ggplot(AAA,aes(GeneRatio,forcats::fct_reorder(Description,Count)))+

## 画出点图

geom_point(aes(color=pvalue, size = Count)) +

## 调整颜色，guide_colorbar调整色图的方向

scale_color_continuous(low="red", high="blue", guide=guide_colorbar(reverse=TRUE))+

## 调整泡泡的大小

scale_size_continuous(range=c(3, 8))+

## 如果用ylab("")或出现左侧空白

labs(y=NULL) +

## 如果没有这一句，上方会到顶

ggtitle("")+

## 设定主题

theme_bw() +

theme(axis.text.x = element_text(colour = "black",

size = font.size, vjust =1 ),

axis.text.y = element_text(colour = "black",

size = font.size, hjust =1 ),

axis.title = element_text(margin=margin(10, 5, 0, 0),

color = "black",size = font.size),

axis.title.y = element_text(angle=90))

p1<-p1 + scale_y_discrete(labels=function(x) str_wrap(x, width=50))

ggsave(filename ='Go_result_BP2.pdf',width = 8,height = 8)

ggsave(filename ='Go_result_BP2.TIFF',width = 8,height = 8)

#用P值来排序，不用padjust

#首先，我获取了富集对象x中的数据框，这是S4对象，用@符号来获取,有293行，是没有筛选过的数据

y =Go_result_CC@result

#我们的横坐标有问题，是因为这里的GeneRatio是字符串，我们现在要把它变成一个数值

## 分别后去分号前面和后面的数，并变成数值

forward <- as.numeric(sub("/\\d+$", "", y$GeneRatio))

backward <- as.numeric(sub("^\\d+/", "", y$GeneRatio))

## 增加数值表示的一列GeneRatio

y$GeneRatio = forward/backward

showCategory =20

#再设定一个字体大小，大小后期可以调整

font.size =12

library(ggplot2)

library(forcats)

library(dplyr)

#复现气泡图

AAA=y %>%

## 安装p值排序，选区既定数目的行

arrange(pvalue) %>%

slice(1:showCategory)

## 开始ggplot2 作图，其中fct_reorder调整因子level的顺序

p1=ggplot(AAA,aes(GeneRatio,forcats::fct_reorder(Description,Count)))+

## 画出点图

geom_point(aes(color=pvalue, size = Count)) +

## 调整颜色，guide_colorbar调整色图的方向

scale_color_continuous(low="red", high="blue", guide=guide_colorbar(reverse=TRUE))+

## 调整泡泡的大小

scale_size_continuous(range=c(3, 8))+

## 如果用ylab("")或出现左侧空白

labs(y=NULL) +

## 如果没有这一句，上方会到顶

ggtitle("")+

## 设定主题

theme_bw() +

theme(axis.text.x = element_text(colour = "black",

size = font.size, vjust =1 ),

axis.text.y = element_text(colour = "black",

size = font.size, hjust =1 ),

axis.title = element_text(margin=margin(10, 5, 0, 0),

color = "black",size = font.size),

axis.title.y = element_text(angle=90))

p1<-p1 + scale_y_discrete(labels=function(x) str_wrap(x, width=50))

ggsave(filename ='Go_result_CC2.pdf',width = 8,height = 8)

ggsave(filename ='Go_result_CC2.TIFF',width = 8,height = 8)

#GO结果画有序条形图

library(ggpubr)

library(tidyverse)

GODATA<-go@result

GODATA$"-log10(Pvalue)"<- -log10(GODATA$pvalue)

GODATA$yyy<- -log10(GODATA$pvalue)

colnames(GODATA)

write.csv(GODATA,"GODATA.csv",row.names =F)#导出结果至默认路径下

#分别选取BP MF CC 的前十个

aaa<-filter(GODATA,ONTOLOGY=='BP')

aaaa<-aaa[1:10,]

bbb<-filter(GODATA,ONTOLOGY=='CC')

bbbb<-bbb[1:10,]

ccc<-filter(GODATA,ONTOLOGY=='MF')

cccc<-ccc[1:10,]

drawdata<-rbind(aaaa,bbbb,cccc)

ggbarplot(drawdata, x = "Description", y = 'yyy',

fill = "ONTOLOGY", # change fill color by cyl

color = "white", # Set bar border colors to white

palette = c('#5FB404','#01DFD7','#C238E5'), # jco journal color palett. see ?ggpar

sort.val = "desc", # Sort the value in dscending order

sort.by.groups = FALSE, # Don't sort inside each group

x.text.angle = 90 # Rotate vertically x axis texts

)

ggbarplot(drawdata, x = "Description", y = "yyy",

fill = "ONTOLOGY", # change fill color by cyl

color = "white", # Set bar border colors to white

palette = c('#5FB404','#12B5EC','#C238E5'), # jco journal color palett. see ?ggpar

sort.val = "asc", # Sort the value in dscending order

sort.by.groups = TRUE, # Sort inside each group

x.text.angle = 75, # Rotate vertically x axis texts

ylab = '-log10(P-Value)',

xlab = 'Pathway'

)

ggsave(filename ='GO三合一2.pdf',width = 7.23,height = 8)

ggsave(filename ='GO三合一2.TIFF',width = 7.23,height = 8)

1. **Analysis of genetic differences is presented separately**

rm(list = ls())

library(limma)

library(ggrepel)

library(ggthemes)

library(tidyverse)

library(pheatmap)

Sys.setenv(LANGUAGE = "en") #显示英文报错信息

options(stringsAsFactors = FALSE) #禁止chr转成factor

# load("FEgenedata.RDATA")

# express.norm <-aaa

# group_list <- read.table("merge.clin.txt", sep = "\t", header = T)

FE<-read.table("O-GlcNAcylation.txt",header=T,sep="\t")

FEgene<-FE[,1]

dat<-read.csv("merge矩阵new.csv",header = T,sep = ",",row.names = 1)

group_list <- read.table("clinicaldata.txt", sep = "\t", header = T)

range(dat)

ccc=intersect(group_list$Accession,colnames(dat))

dat=dat[,ccc]

aaa<-intersect(rownames(dat),FEgene)

express.norm <-dat[aaa,]

x<-read.csv("差异分析结果.csv",header = T,row.names = 1)

x<-x[aaa,]

write.csv(x, "merge差异分析结果.交集基因.csv", quote = F)

p.cut<-0.05

logFC.cut<-1

volcano<-x

volcano$type[(volcano$adj.P.Val > p.cut|volcano$adj.P.Val=="NA")|(volcano$logFC < logFC.cut)& volcano$logFC > -logFC.cut] <- "none significant"

volcano$type[volcano$adj.P.Val <= p.cut & volcano$logFC >= logFC.cut] <- "up-regulated"

volcano$type[volcano$adj.P.Val <= p.cut & volcano$logFC <= -logFC.cut] <- "down-regulated"

p = ggplot(volcano,aes(logFC,-1*log10(adj.P.Val),color=type))

p + geom_point()

x_lim <- max(volcano$logFC,-x$logFC)

gg=p + geom_point( aes(size = abs(logFC)),alpha = 0.4) + xlim(-x_lim,x_lim) +labs(x="log2(FC)",y="-log10(adj.P.Val)")+

scale_color_manual(values =c("blue","grey","red"))+

geom_hline(aes(yintercept=-1*log10(p.cut)),colour="black", linetype="dashed") +

geom_vline(xintercept=c(-logFC.cut,logFC.cut),colour="black", linetype="dashed")

print(gg)

pdf(file="merge火山图.PDF", width=6, height=6)

gg

dev.off()

#热图，选取高低表达top10

volcano1<-dplyr::filter(volcano,adj.P.Val < 0.05)

genename<-rownames(volcano1)

express1<-as.data.frame(express.norm)

express1$ID<-rownames(express1)

phonedata<-dplyr::filter(express1,ID %in% genename)

phonedata<-dplyr::select(phonedata,-ID)

group_list<-group_list[order(group_list$condition,decreasing = T),]

group_list[,2]

annotation_col<-data.frame(group_list[,2])

rownames(annotation_col)<-group_list[,1]

colnames(annotation_col)<-"Group"

phonedata<-select(phonedata,rownames(annotation_col))

pheatmap(phonedata,scale = "row",cluster_row=T,cluster_col=F, annotation_col = annotation_col,show_colnames = F

,border_color = F,color = colorRampPalette(c("blue", "white", "red"))(50))

dev.off()

pdf(file="merge热图.PDF", width=8, height=10)

pheatmap(phonedata,scale = "row",cluster_row=T,cluster_col=F, annotation_col = annotation_col,show_colnames = F

,border_color = F,color = colorRampPalette(c("blue", "white", "red"))(50))

dev.off()

1. **Machine learning**

library(glmnet)

library(tidyverse)

rm(list = ls())

set.seed(1234)

data=read.csv("merge矩阵new.csv",header = T,sep = ",",row.names = 1)

gene=read.table("差异基因.txt",header = T,sep = "\t")

gene=gene[,1]

data=data[gene,]

data=as.data.frame(t(data))

data$ID=rownames(data)

clin=read.table("clinicaldata.txt",header = T,sep = "\t")

colnames(clin)[1]="ID"

aaa=intersect(clin$ID,data$ID)

clin=dplyr::filter(clin, ID %in% aaa)

data=dplyr::filter(data, ID %in% aaa)

data1=inner_join(clin,data,by = "ID")

rt=dplyr::select(data1,gene)

rownames(rt)=data1$ID

#构建模型

x=as.matrix(rt)

y=data1$condition

fit=glmnet(x, y, family = "binomial", alpha=1)

pdf(file="fit.pdf",width=6,height=5.5)

plot(fit)

dev.off()

cvfit=cv.glmnet(x, y, family="binomial", alpha=1,type.measure='deviance',nfolds = 10)

pdf(file="cvfit.pdf",width=6,height=5.5)

plot(cvfit)

dev.off()

#输出筛选的特征基因

coef=coef(fit, s = cvfit$lambda.min)

coef

#提取选中的基因名

active.min = which(coef != 0)-1

active.min = active.min[-1]

geneids <- colnames(x)[active.min]

geneids

#提取选中的基因对应的coefficient

index.min = coef[active.min+1]

index.min

combine <- cbind(geneids, index.min)#合并基因名和coef

colnames(combine)=c("ID","risk")

combine=as.data.frame(combine)

write.csv(combine,"risk.csv",row.names = F)

index=which(coef != 0)

lassoGene=row.names(coef)[index]

lassoGene=lassoGene[-1]

write.table(lassoGene, file="LASSO.gene.txt", sep="\t", quote=F, row.names=F, col.names=F)

#引用包

library(e1071)

library(kernlab)

library(caret)

set.seed(1234)

#读取输入文件

data=x

group=y

#SVM-RFE分析

Profile=rfe(x=data,

y=as.numeric(as.factor(group)),

sizes = c(2,4,6,8, seq(10,40,by=3)),

rfeControl = rfeControl(functions = caretFuncs, method = "cv"),

methods="svmRadial")

#绘制图形

pdf(file="SVM-RFE.pdf", width=6, height=5.5)

par(las=1)

x = Profile$results$Variables

y = Profile$results$RMSE

plot(x, y, xlab="Variables", ylab="RMSE (Cross-Validation)", col="darkgreen")

lines(x, y, col="darkgreen")

#标注交叉验证误差最小的点

wmin=which.min(y)

wmin.x=x[wmin]

wmin.y=y[wmin]

points(wmin.x, wmin.y, col="blue", pch=16)

text(wmin.x, wmin.y, paste0('N=',wmin.x), pos=2, col=2)

dev.off()

#输出选择的基因

featureGenes=Profile$optVariables

write.table(file="SVM-RFE.gene.txt", featureGenes, sep="\t", quote=F, row.names=F, col.names=F)

#随机森林

set.seed(1234)

data1=arrange(data1,desc(condition))

group=data1$condition

data2=data1

rownames(data2)=data2$ID

data2=data2[,-1]

data2=data2[,-1]

colnames(data2)=str_replace_all(colnames(data2),"-",".")

library(randomForest)

library(ggpubr)

rf=randomForest(as.factor(group)~., data=data2, ntree=500)

pdf(file="forest.pdf", width=6, height=6)

plot(rf, main="Random forest", lwd=2)

dev.off()

#找出误差最小的点

optionTrees=which.min(rf$err.rate[,1])

optionTrees

rf2=randomForest(as.factor(group)~., data=data2, ntree=optionTrees)

#查看基因的重要性

#绘制基因的重要性图

importance=importance(x=rf2)

importance=as.data.frame(importance)

importance$size=rownames(importance)

importance=importance[,c(2,1)]

names(importance)=c("Gene","importance")

importance=arrange(importance,desc(importance))

#展示前10个基因的重要性

af=importance[1:7,]

p=ggdotchart(af, x = "Gene", y = "importance",

color = "importance", # Custom color palette

sorting = "descending", # Sort value in descending order

add = "segments", # Add segments from y = 0 to dots

add.params = list(color = "lightgray", size = 2), # Change segment color and size

dot.size = 6, # Add mpg values as dot labels

font.label = list(color = "white", size = 9,

vjust = 0.5), # Adjust label parameters

ggtheme = theme_bw() , # ggplot2 theme

rotate=TRUE )#翻转坐标轴

p1=p+ geom_hline(yintercept = 0, linetype = 2, color = "lightgray")+

gradient_color(palette =c(ggsci::pal_npg()(2)[2],ggsci::pal_npg()(2)[1]) ) +#颜色

grids()

#保存图片

pdf(file="importance.pdf", width=6, height=8)

print(p1)

dev.off()

#挑选疾病特征基因

rfGenes=importance[order(importance[,"importance"], decreasing = TRUE),]

write.table(rfGenes, file="rfGenes.xls", sep="\t", quote=F, col.names=T, row.names=F)

1. **ssGSEA**

##ssGESA

rm(list = ls())

#引用包

library(reshape2)

library(tidyverse)

library(ggpubr)

library(limma)

library(GSEABase)

library(GSVA)

expFile="merge矩阵new.csv" #表达输入文件

gmtFile="immune.gmt" #免疫数据集文件

clusterFile="clinicaldata.txt" #分型输入文件

#读取表达输入文件,并对输入文件整理

rt=read.csv(expFile, header=T, sep=",", check.names=F)

rt=as.matrix(rt)

rownames(rt)=rt[,1]

exp=rt[,2:ncol(rt)]

dimnames=list(rownames(exp),colnames(exp))

data=matrix(as.numeric(as.matrix(exp)),nrow=nrow(exp),dimnames=dimnames)

data=avereps(data)

#读取基因集文件

geneSets=getGmt(gmtFile, geneIdType=SymbolIdentifier())

#ssGSEA分析

ssgseaScore=gsva(data, geneSets, method='ssgsea', kcdf='Gaussian', abs.ranking=TRUE)

#对ssGSEA打分进行矫正

normalize=function(x){

return((x-min(x))/(max(x)-min(x)))}

ssgseaScore=normalize(ssgseaScore)

#输出ssGSEA打分结果

ssgseaOut=rbind(id=colnames(ssgseaScore), ssgseaScore)

write.table(ssgseaOut,file="merge_ssGSEA.result.txt",sep="\t",quote=F,col.names=F)

#读取分型文件

cluster=read.table(clusterFile, header=T, sep="\t", check.names=F, row.names=1)

#数据合并

ssgseaScore=t(ssgseaScore)

sameSample=intersect(row.names(ssgseaScore), row.names(cluster))

ssgseaScore=ssgseaScore[sameSample,,drop=F]

cluster=cluster[sameSample,,drop=F]

scoreCluster=cbind(ssgseaScore, cluster)

colnames(scoreCluster)<-str_replace_all(colnames(scoreCluster),"na","")

#把数据转换成ggplot2输入文件

data=melt(scoreCluster, id.vars=c("condition"))

colnames(data)=c("cluster", "Immune", "Fraction")

#绘制箱线图

bioCol=c("#0066FF","#FF9900","#FF0000","#6E568C","#7CC767","#223D6C","#D20A13","#FFD121","#088247","#11AA4D")

bioCol=bioCol[1:length(levels(factor(data[,"cluster"])))]

library(tidyverse)

data$Immune=str_replace_all(data$Immune,"na","")

p=ggboxplot(data, x="Immune", y="Fraction", color="cluster",

ylab="Immune infiltration",

xlab="",

legend.title="cluster",

palette=bioCol)

p=p+rotate_x_text(50)

pdf(file="merge_ssGSEA_boxplot.pdf", width=8, height=6.5) #输出图片文件

p+stat_compare_means(aes(group=cluster),symnum.args=list(cutpoints = c(0, 0.001, 0.01, 0.05, 1), symbols = c("***", "**", "*", "ns")),label = "p.signif")

dev.off()

colnames(scoreCluster)<-str_replace_all(colnames(scoreCluster),"na","")

data <- scoreCluster

data=dplyr::select(data,-"condition")

library(corrplot)

#相关性矩阵

M=cor(data)

res1=cor.mtest(data, conf.level = 0.95)

#绘制相关性图形

pdf(file="merge_cor.pdf", width=8, height=8)

corrplot(M,

order="original",

method = "circle",

type = "upper",

tl.cex=0.8, pch=T,

p.mat = res1$p,

insig = "label_sig",

pch.cex = 1.6,

sig.level=0.05,

number.cex = 1,

col=colorRampPalette(c("blue", "white", "red"))(50),

tl.col="black")

1. **Gene and immune cell correlation**

rm(list = ls())

library(circlize)

library(ggsci)

library(parallel)

library(tidyverse)

Sys.setenv(LANGUAGE = "en") #显示英文报错信息

options(stringsAsFactors = FALSE) #禁止chr转成factor

# 计算相关系数的函数

genecor.parallel <- function(data,gene,cl){

cl <- makeCluster(cl)

y <- as.numeric(data[gene,])

rownames <- rownames(data)

dataframes <- do.call(rbind, parLapply(cl=cl,rownames, function(x){

dd <- cor.test(as.numeric(data[x,]), y, type="spearman")

data.frame(Gene_1=gene, Gene_2=x, cor=dd$estimate, p.value=dd$p.value)

}))

stopCluster(cl)

return(dataframes)

}

# 画图的函数

genecor_circleplot <- function(x){

Corr <- data.frame(rbind(data.frame(Gene=x[,1], Correlation=x[,3]),

data.frame(Gene=x[,2], Correlation=x[,3])), stringsAsFactors = F)

Corr$Index <- seq(1,nrow(Corr),1) #记录基因的原始排序，记录到Index列

Corr <- Corr[order(Corr[,1]),] #按照基因名排序

corrsp <- split(Corr,Corr$Gene)

corrspe <- lapply(corrsp, function(x){x$Gene_Start<-0

#依次计算每个基因的相关系数总和，作为基因终止位点

if (nrow(x)==1){x$Gene_End<-1}else{

x$Gene_End<-sum(abs(x$Correlation))}

x})

GeneID <- do.call(rbind,corrspe)

GeneID <- GeneID[!duplicated(GeneID$Gene),]

#基因配色

mycol <- pal_d3("category20c")(20)

n <- nrow(GeneID)

GeneID$Color <- mycol[1:n]

#连线的宽度是相关系数的绝对值

Corr[,2] <- abs(Corr[,2])

corrsl <- split(Corr,Corr$Gene)

aaaaa <- c()

corrspl <- lapply(corrsl,function(x){nn<-nrow(x)

for (i in 1:nn){

aaaaa[1] <- 0

aaaaa[i+1] <- x$Correlation[i]+aaaaa[i]}

bbbbb <- data.frame(V4=aaaaa[1:nn],V5=aaaaa[2:(nn+1)])

bbbbbb <- cbind(x,bbbbb)

bbbbbb

})

Corr <- do.call(rbind,corrspl)

#根据Index列，把基因恢复到原始排序

Corr <- Corr[order(Corr$Index),]

#V4是起始位置，V5是终止位置

#把它写入Links里，start_1和end_1对应Gene_1，start_2和end_2对应Gene_2

x$start_1 <- Corr$V4[1:(nrow(Corr)/2)]

x$end_1 <- Corr$V5[1:(nrow(Corr)/2)]

x$start_2 <- Corr$V4[(nrow(Corr)/2 + 1):nrow(Corr)]

x$end_2 <- Corr$V5[(nrow(Corr)/2 + 1):nrow(Corr)]

#连线（相关系数）的配色

#相关系数最大为1，最小-1，此处设置201个颜色

#-1到0就是前100，0到1就是后100

color <- data.frame(colorRampPalette(c("#67BE54", "#FFFFFF", "#F82C2B"))(201))

#根据相关系数的数值，给出相应的颜色

for (i in 1:nrow(x)){

x[i,8] <- substring(color[x[i,3] * 100 + 101, 1], 1, 7)

}

names(x)[8] <- "color"

#绘图区设置

#par(mar=rep(0,4))

circos.clear()

circos.par(start.degree = 90, #从哪里开始画，沿着逆时针顺序

gap.degree = 5, #基因bar之间的间隔大小

track.margin = c(0,0.23), #值越大，基因跟连线的间隔越小

cell.padding = c(0,0,0,0)

)

circos.initialize(factors = GeneID$Gene,

xlim = cbind(GeneID$Gene_Start, GeneID$Gene_End))

#先画基因

circos.trackPlotRegion(ylim = c(0, 1), factors = GeneID$Gene,

track.height = 0.05, #基因线条的胖瘦

panel.fun = function(x, y) {

name = get.cell.meta.data("sector.index")

i = get.cell.meta.data("sector.numeric.index")

xlim = get.cell.meta.data("xlim")

ylim = get.cell.meta.data("ylim")

circos.text(x = mean(xlim), y = 1,

labels = name,

cex = 1, #基因ID文字大小

niceFacing = TRUE, #保持基因名的头朝上

facing = "bending", #基因名沿着圆弧方向，还可以是reverse.clockwise

adj = c(0.5, -2.8), #基因名所在位置，分别控制左右和上下

font = 2 #加粗

)

circos.rect(xleft = xlim[1],

ybottom = ylim[1],

xright = xlim[2],

ytop = ylim[2],

col = GeneID$Color[i],

border = GeneID$Color[i])

circos.axis(labels.cex = 0.7,

direction = "outside"

)})

#画连线

for(i in 1:nrow(x)){

circos.link(sector.index1 = x$Gene_1[i],

point1 = c(x[i, 4], x[i, 5]),

sector.index2 = x$Gene_2[i],

point2 = c(x[i, 6], x[i, 7]),

col = paste(x$color[i], "C9", sep = ""),

border = FALSE,

rou = 0.7

)}

#画图例

i <- seq(0,0.995,0.005)

rect(-1+i/2, #xleft

-1, #ybottom

-0.9975+i/2, #xright

-0.96, #ytop

col = paste(as.character(color[,1]), "FF", sep = ""),

border = paste(as.character(color[,1]), "FF", sep = ""))

text(-0.97, -1.03, "-1")

text(-0.51, -1.03, "1")

}

inputtemp <- read.csv("merge矩阵new.csv", header = T,sep = "," ,row.names = 1)

aaa<-read.table("机器学习交集.txt",header = T)

aaa<-aaa[,1]

inputtemp<-as.data.frame(t(inputtemp))

inputtemp<-dplyr::select(inputtemp,aaa)

clin<-read.table("clinicaldata.txt",header = T)

inputtemp=inputtemp[clin$Accession,]

ccc<-inputtemp

ddd<-clin

colnames(ddd)[1]="ID"

ccc$ID<-rownames(ccc)

eee<-inner_join(ddd,ccc,by="ID")

write.table(eee,file = "mergeROC.txt",row.names = F,sep = "\t")

genecorl <- lapply(colnames(inputtemp),function(x){

ddd <- genecor.parallel(data = t(inputtemp), cl=1, gene=x) #一定要注意cl参数根据自己电脑cpu线程调整

ddd

})

genecor <- do.call(rbind, genecorl)

# 删掉p value = 0的，也就是自己跟自己配对

genecorr <- genecor[-which(genecor$p.value==0),]

# 删掉A vs B 和 B vs A其中一个

genecorrr<-genecorr[!duplicated(genecorr$cor),]

# 保存到文件

genecorrr$p.value <- NULL

genecor_circleplot(genecorrr)

pdf("mergecorrelation.pdf", width = 5, height = 5)

genecor_circleplot(genecorrr)

dev.off()

drawdata=eee

library(tidyverse)

library(corrplot)

library(circlize)

Sys.setenv(LANGUAGE = "en") #显示英文报错信息

options(stringsAsFactors = FALSE) #禁止chr转成factor

inputtemp1 <- read.table("merge_ssGSEA.result.txt", header=T,sep = "\t" )

rownames(inputtemp1)=inputtemp1$id

rownames(inputtemp1)=str_replace_all(rownames(inputtemp1),"na","")

inputtemp1=inputtemp1[,-1]

inputtemp1=as.data.frame(t(inputtemp1))

inputtemp1$ID=rownames(inputtemp1)

inputtemp$ID<-rownames(inputtemp)

drawdata<-inner_join(inputtemp1,inputtemp,by="ID")

gene <- "SEMA5A"

y <- as.numeric(drawdata[,gene])

drawdata1<-select(drawdata,2:23)

### 第1,写出单次处理的function

mycor = function(x){

dd = cor.test(as.numeric(drawdata1[, x]),y,method ="spearman",exact=FALSE)

data.frame(cell=x,cor=dd$estimate,p.value=dd$p.value)

}

colnames(drawdata1)<-gsub("\\."," ",colnames(drawdata1))

### 第2步lapply批量作用于函数，返回list

lapplylist = lapply(colnames(drawdata1),mycor)

### 第3步do.call 转换list

cor_data <- do.call(rbind,lapplylist)

cor_data %>%

filter(p.value <0.05) %>%

ggplot(aes(cor,forcats::fct_reorder(cell,cor)))+

geom_segment(aes(xend=0,yend=cell))+

geom_point(aes(col=p.value,size=abs(cor)))+

scale_colour_gradientn(colours=c("#7fc97f","#984ea3"))+

#scale_color_viridis_c(begin = 0.5, end = 1)+

scale_size_continuous(range =c(2,8))+

theme_bw()+

ylab(NULL)+

xlab(gene)

write.csv(cor_data,file = paste0(gene,"相关性分析结果.csv"))

pdf(file=paste0(gene,"免疫细胞相关性分析.pdf"),width=5,height=7)

cor_data %>%

filter(p.value <0.05) %>%

ggplot(aes(cor,forcats::fct_reorder(cell,cor)))+

geom_segment(aes(xend=0,yend=cell))+

geom_point(aes(col=p.value,size=abs(cor)))+

scale_colour_gradientn(colours=c("#7fc97f","#984ea3"))+

#scale_color_viridis_c(begin = 0.5, end = 1)+

scale_size_continuous(range =c(2,8))+

theme_bw()+

ylab(NULL)+

xlab(gene)

dev.off()

1. **ROC**

rm(list = ls())

df <- read.table("mergeROC.txt",head=T,sep="\t",check.names = F)

head(df)

df<-df[,-1]

library("pROC")

#定义足够多的颜色，后面画线时从这里选颜色

mycol <- c("slateblue","seagreen3","dodgerblue","firebrick1","lightgoldenrod","magenta","orange2","grey","green","red")

pdf("ROC.pdf",height=6,width=6)

auc.out <- c()

#先画第一条线，此处是miRNA1

x <- plot.roc(df[,1],df[,2],ylim=c(0,1),xlim=c(1,0),

smooth=F, #绘制平滑曲线

ci=TRUE,

main="",

#print.thres="best", #把阈值写在图上，其sensitivity+ specificity之和最大

col=mycol[2],#线的颜色

lwd=2, #线的粗细

legacy.axes=T)#采用大多数paper的画法，横坐标是“1-specificity”，从0到1

ci.lower <- round(as.numeric(x$ci[1]),3) #置信区间下限

ci.upper <- round(as.numeric(x$ci[3]),3) #置信区间上限

auc.ci <- c(colnames(df)[2],round(as.numeric(x$auc),3),paste(ci.lower,ci.upper,sep="-"))

auc.out <- rbind(auc.out,auc.ci)

#再用循环画第二条和后面更多条曲线

for (i in 3:ncol(df)){

x <- plot.roc(df[,1],df[,i],

add=T, #向前面画的图里添加

smooth=F,

ci=TRUE,

col=mycol[i],

lwd=2,

legacy.axes=T)

ci.lower <- round(as.numeric(x$ci[1]),3)

ci.upper <- round(as.numeric(x$ci[3]),3)

auc.ci <- c(colnames(df)[i],round(as.numeric(x$auc),3),paste(ci.lower,ci.upper,sep="-"))

auc.out <- rbind(auc.out,auc.ci)

}

# 输出AUC、AUC CI到文件

auc.out <- as.data.frame(auc.out)

colnames(auc.out) <- c("Name","AUC","AUC CI")

#绘制图例

legend.name <- paste(colnames(df)[2:length(df)],"AUC",auc.out$AUC,sep=" ")

legend("bottomright",

legend=legend.name,

col = mycol[2:length(df)],

lwd = 2,

bty="n")

dev.off()

**9.GSEA**

library(tidyverse)

library(ggpubr)

library(survminer)

library(survival)

library(export)

library(survivalROC)

library(pheatmap)

library(data.table)

library(org.Hs.eg.db)

library(clusterProfiler)

library(biomaRt)

library(enrichplot)

library(ReactomePA)

library(igraph)

library(ggraph)

library(ggradar)

rm(list = ls())

FF<-read.csv("merge矩阵new.csv",sep = ",",header = T)

rownames(FF)<-FF[,1]

FF<-FF[,-1]

FF<-as.data.frame(t(FF))

gene = "SEMA5A"

y <- as.numeric(FF[,gene])#开始相关性分析

colnames <- colnames(FF)

cor_data_df <- data.frame(colnames)

for (i in 1:length(colnames(FF))){

test <- cor.test(as.numeric(FF[,i]),y,type="pearson")

cor_data_df[i,2] <- test$estimate

cor_data_df[i,3] <- test$p.value

}

names(cor_data_df) <- c("symbol","correlation","pvalue")

write.csv (cor_data_df, file =paste(gene,'批量相关性分析.csv',sep=' '), row.names =FALSE)#将文件导出

#相关性分析结果排序选取P<0.05,top50的做相关性热图，R>0.3的做相关性图

cor_data_df<-na.omit(cor_data_df)

pos<-cor_data_df %>%

filter(pvalue < 0.05 ) %>%

arrange(desc(correlation)) %>%

top_n(50,correlation)

neg<-cor_data_df %>%

filter(pvalue < 0.05 ) %>%

arrange(correlation) %>%

top_n(-49,correlation)

#正相关热图

phemapdata<-FF %>%

dplyr::select(pos$symbol)

phemapdata<-arrange(phemapdata,desc(phemapdata[,gene]))

phemapdata<-as.data.frame(t(phemapdata))

p<-pheatmap(phemapdata,scale="row",cluster_row=F,cluster_col=F,legend= T,show_colnames = F,color = colorRampPalette(c("blue", "white", "red"))(50))

dev.off()

pdf(file = paste(gene,'正相关性热图.pdf',sep=' '),width = 8,height = 6)

print(p)

dev.off()

#负相关热图

phemapdata<-FF %>%

dplyr::select(gene,neg$symbol)

phemapdata<-arrange(phemapdata,desc(phemapdata[,gene]))

phemapdata<-as.data.frame(t(phemapdata))

p<-pheatmap(phemapdata,scale="row",cluster_row=F,cluster_col=F,legend= T,show_colnames = F,color = colorRampPalette(c("blue", "white", "red"))(50))

dev.off()

pdf(file = paste(gene,'负相关性热图.pdf',sep=' '),width = 8,height = 6)

print(p)

dev.off()

FUJI<-cor_data_df %>%

filter(pvalue < 0.05 ) %>%

arrange(desc(correlation)) %>%

top_n(500,correlation)

genename <- as.character(FUJI[,1]) #提取第一列基因名

gene_map <- biomaRt::select(org.Hs.eg.db, keys=genename, keytype="SYMBOL", columns=c("ENTREZID"))

genelist_input<-gene_map[,2]

genelist_input<-na.omit(genelist_input)

#GO分析

#BP

Go_result_BP <- enrichGO(genelist_input, 'org.Hs.eg.db', ont="BP", pvalueCutoff=1) #基因ID类型为ENSEMBL的ID形式，选择BP功能组，以P值0.05为界限

p1<-dotplot(Go_result_BP, showCategory=20)

p2<-barplot(Go_result_BP, showCategory=20)

p1<-p1 + scale_y_discrete(labels=function(x) str_wrap(x, width=50))

p2<-p2 + scale_x_discrete(labels=function(x) str_wrap(x, width=50))

y=Go_result_BP

yy<-as.data.frame(y)

write.csv(as.data.frame(y),paste(gene,"GO-BP.csv",sep = " "),row.names =F)#导出结果至默认路径下。

if(length(rownames(yy))!= 0) {

ggsave(p1,filename = paste(gene,'富集分析GO_BP气泡图.pdf',sep=' '),width = 7.23,height = 8)

ggsave(p1,filename = paste(gene,'富集分析GO_BP气泡图.TIFF',sep=' '),width = 7.23,height = 8)

}

#CC

Go_result_CC <- enrichGO(genelist_input, 'org.Hs.eg.db', ont="CC", pvalueCutoff=1) #基因ID类型为ENSEMBL的ID形式，选择CC功能组，以P值0.05为界限

p1<-dotplot(Go_result_CC, showCategory=20) #气泡图，显示前二十个

p2<-barplot(Go_result_CC, showCategory=20) #条形图，显示前二十个

p1<-p1 + scale_y_discrete(labels=function(x) str_wrap(x, width=50))

p2<-p2 + scale_x_discrete(labels=function(x) str_wrap(x, width=50))

y=Go_result_CC

yy<-as.data.frame(y)

write.csv(as.data.frame(y),paste(gene,"GO-CC.csv",sep = " "),row.names =F)#导出结果至默认路径下

if(length(rownames(yy))!= 0) {

ggsave(p1,filename = paste(gene,'富集分析GO_CC气泡图.pdf',sep=' '),width = 7.23,height = 8)

ggsave(p1,filename = paste(gene,'富集分析GO_CC气泡图.TIFF',sep=' '),width = 7.23,height = 8)

}

#MF

Go_result_MF <- enrichGO(genelist_input, 'org.Hs.eg.db',ont="MF", pvalueCutoff=1) #基因ID类型为ENSEMBL的ID形式，选择MF功能组，以P值0.05为界限

p1<-dotplot(Go_result_MF, showCategory=20) #气泡图，显示前二十个

p2<-barplot(Go_result_MF, showCategory=20) #条形图，显示前二十个

p1<-p1 + scale_y_discrete(labels=function(x) str_wrap(x, width=50))

p2<-p2 + scale_x_discrete(labels=function(x) str_wrap(x, width=50))

y=Go_result_MF

write.csv(as.data.frame(y),file = paste(gene,"GO-MF.csv",sep = " "),row.names =F)#导出结果至默认路径下

yy<-as.data.frame(y)

if(length(rownames(yy))!= 0){

ggsave(p1,filename = paste(gene,'富集分析GO_MF气泡图.pdf',sep=' '),width = 7.23,height = 8)

ggsave(p1,filename = paste(gene,'富集分析GO_MF气泡图.TIFF',sep=' '),width = 7.23,height = 8)

}

#KEGG分析

KEGG_result <- enrichKEGG(genelist_input, keyType = "kegg",pvalueCutoff=1,qvalueCutoff=1,pAdjustMethod = "BH", minGSSize = 5, maxGSSize = 500,organism = "hsa", use_internal_data=T) #KEGG富集分析

p1<-barplot(KEGG_result, showCategory=20)#绘制条形图

p2<-dotplot(KEGG_result, showCategory=20) #气泡图，显示前二十个

p1<-p1 + scale_x_discrete(labels=function(x) str_wrap(x, width=50))

p2<-p2 + scale_y_discrete(labels=function(x) str_wrap(x, width=50))

y=KEGG_result

yy<-as.data.frame(y)

write.csv(as.data.frame(y), file=paste(gene,"KEGG.csv",sep = " "),row.names =F)#导出结果至默认路径下

if(length(rownames(yy))!= 0){

ggsave(p2,filename = paste(gene,'富集分析KEGG气泡图.pdf',sep=' '),width = 7.23,height = 8)

ggsave(p2,filename = paste(gene,'富集分析KEGG气泡图.TIFF',sep=' '),width = 7.23,height = 8)

}

#GSEA分析

GSEAdata<-cor_data_df %>%

filter(pvalue < 0.05 ) %>%

arrange(desc(correlation)) %>%

dplyr::select(symbol,correlation)

genename <- as.character(GSEAdata[,1]) #提取第一列基因名

gene_map <- biomaRt::select(org.Hs.eg.db, keys=genename, keytype="SYMBOL", columns=c("ENTREZID"))

colnames(gene_map)[1]<- 'symbol'

genelist_input<-gene_map %>%

na.omit() %>%

inner_join(GSEAdata,by= 'symbol') %>%

dplyr::select(ENTREZID,correlation)

geneList = genelist_input[,2]

names(geneList) = as.character(genelist_input[,1])

geneList = sort(geneList, decreasing = TRUE)

#GSEA分析——GO

Go_gseresult <- gseGO(geneList, org.Hs.eg.db, keyType = "ENTREZID", ont="all", nPerm = 1000, minGSSize = 10, maxGSSize = 1000, pvalueCutoff=1)

#GSEA分析——KEGG

KEGG_gseresult <- gseKEGG(geneList, nPerm = 1000, minGSSize = 10, maxGSSize = 1000, pvalueCutoff=1,use_internal_data=T)

#GSEA分析——Reactome

Go_Reactomeresult <- gsePathway(geneList, nPerm = 1000, minGSSize = 10, maxGSSize = 1000, pvalueCutoff=1)

write.csv (Go_gseresult, file = paste(gene,'GSEA_GO.csv',sep=' '), row.names =F)

save(Go_gseresult,file = paste(gene,'GSEA_GO.RDATA',sep=' '))

write.csv (KEGG_gseresult, file = paste(gene,'GSEA_KEGG.csv',sep=' '), row.names =F)

save(KEGG_gseresult,file = paste(gene,'GSEA_KEGG.RDATA',sep=' '))

write.csv (Go_Reactomeresult,file = paste(gene,'GSEA_Reactome.csv',sep=' '), row.names =F)

save(Go_Reactomeresult,file = paste(gene,'GSEA_Reactome.RDATA',sep=' '))

#波浪图

p<-ridgeplot(Go_gseresult,20) #输出前十个结果

p<-p + scale_y_discrete(labels=function(x) str_wrap(x, width=50))

ggsave(p,filename = paste(gene,'GSEA_GO波浪图.pdf',sep=' '),width = 8,height = 8)

ggsave(p,filename = paste(gene,'GSEA_GO波浪图.TIFF',sep=' '),width = 8,height = 8)

p<-ridgeplot(KEGG_gseresult, 20) #输出前十个结果

p<-p + scale_y_discrete(labels=function(x) str_wrap(x, width=50))

ggsave(p,filename = paste(gene,'GSEA_KEGG波浪图.pdf',sep=' '),width = 8,height = 8)

ggsave(p,filename = paste(gene,'GSEA_KEGG波浪图.TIFF',sep=' '),width = 8,height = 8)

p<-ridgeplot(Go_Reactomeresult, 20) #输出前十个结果

p<-p + scale_y_discrete(labels=function(x) str_wrap(x, width=50))

ggsave(p,filename = paste(gene,'GSEA_Reactomeresult波浪图.pdf',sep=' '),width = 8,height = 8)

ggsave(p,filename = paste(gene,'GSEA_Reactomeresult波浪图.TIFF',sep=' '),width = 8,height = 8)

**10.Unsupervised clustering of genes**

rm(list = ls())

load("merge_UC.RDATA")

Merge<-outTab1

genename<-read.table("机器学习交集.txt",header = T,sep = "\t")

AAA<-intersect(genename[,1],rownames(Merge))

geneexpr<-Merge[AAA,]

write.table(geneexpr,"geneexpr.txt",quote = F,row.names =T ,sep = "\t")

save(geneexpr,file = "geneexpr.RDATA")

library(ConsensusClusterPlus)

#读取输入文件

data=geneexpr

data=as.matrix(data)

#聚类

maxK=9

results=ConsensusClusterPlus(data,

maxK=maxK,

reps=50,

pItem=0.8,

pFeature=1,

clusterAlg="pam",

distance="euclidean",

seed=123456,

plot="png")

#输出分型结果

clusterNum=2 #分几类，根据判断标准判断

cluster=results[[clusterNum]][["consensusClass"]]

cluster=as.data.frame(cluster)

colnames(cluster)=c("cluster")

letter=c("A","B","C","D","E","F","G")

uniqClu=levels(factor(cluster$cluster))

cluster$cluster=letter[match(cluster$cluster, uniqClu)]

clusterOut=rbind(ID=colnames(cluster), cluster)

write.table(clusterOut, file="Cluster.txt", sep="\t", quote=F, col.names=F)

1. **4 hub genes between subtypes**

rm(list = ls())

#引用包

library(limma)

library(reshape2)

library(ggpubr)

expFile="geneexpr.txt" #表达输入文件

geneCluFile="Cluster.txt" #基因分型文件

#读取表达输入文件

rt=read.table(expFile, header=T, sep="\t", check.names=F)

# rownames(rt)=rt[,1]

# rt<-rt[,-1]

rt=as.matrix(rt)

exp=rt

dimnames=list(rownames(exp),colnames(exp))

data=matrix(as.numeric(as.matrix(exp)),nrow=nrow(exp),dimnames=dimnames)

data=avereps(data)

data=t(data)

#读取基因分型文件

geneClu=read.table(geneCluFile, header=T, sep="\t", check.names=F, row.names=1)

#合并数据

sameSample=intersect(row.names(data), row.names(geneClu))

expClu=cbind(data[sameSample,,drop=F], geneClu[sameSample,,drop=F])

#把数据转换成ggplot2输入文件

data=melt(expClu, id.vars=c("cluster"))

colnames(data)=c("Cluster", "Gene", "Expression")

#设置颜色

bioCol=c("#0066FF","#FF9900","#FF0000","#6E568C","#7CC767","#223D6C","#D20A13","#FFD121","#088247","#11AA4D")

bioCol=bioCol[1:length(levels(factor(data[,"Cluster"])))]

#绘制箱线图

p=ggboxplot(data, x="Gene", y="Expression", color = "Cluster",

ylab="Gene expression",

xlab="",

legend.title="Cluster",

palette = bioCol,

width=1)

p=p+rotate_x_text(60)

p1=p+stat_compare_means(aes(group=Cluster),

symnum.args=list(cutpoints = c(0, 0.001, 0.01, 0.05, 1), symbols = c("***", "**", "*", "ns")),

label = "p.signif",method = "t.test")

#输出箱线图

pdf(file="boxplot.pdf", width=6, height=5)

print(p1)

dev.off()

**12.heatmap between subtypes**

rm(list = ls())

library(pheatmap) #引用包

expFile="geneexpr.txt" #表达输入文件

clusterFile="Cluster.txt" #聚类结果文件

cliFile="clinicaldata.txt" #临床数据文件

#读取输入文件

exp=read.table(expFile, header=T, sep="\t", check.names=F, row.names=1)

exp=t(exp)

cluster=read.table(clusterFile, header=T, sep="\t", check.names=F, row.names=1)

#合并表达和分型数据

sameSample=intersect(row.names(exp), row.names(cluster))

exp=exp[sameSample, , drop=F]

cluster=cluster[sameSample, , drop=F]

expCluster=cbind(exp, cluster)

Project=gsub("(.*?)\\_.*", "\\1", rownames(expCluster))

library(tidyverse)

expCluster=cbind(expCluster, Project)

#合并临床数据

cli=read.table(cliFile, header=T, sep="\t", check.names=F, row.names=1)

sameSample=intersect(row.names(expCluster), row.names(cli))

expCluster=expCluster[sameSample,,drop=F]

cli=cli[sameSample,,drop=F]

data=cbind(expCluster, cli)

#提取热图数据

data=data[order(data$cluster),]

Type=data[,((ncol(exp)+1):ncol(data))]

Type=Type[,-2]

data=t(data[,1:ncol(exp)])

#聚类颜色

bioCol=c("#0066FF","#FF9900","#FF0000","#6E568C","#7CC767","#223D6C","#D20A13","#FFD121","#088247","#11AA4D")

ann_colors=list()

CluCol=bioCol[1:length(levels(factor(Type$cluster)))]

names(CluCol)=levels(factor(Type$cluster))

ann_colors[["cluster"]]=CluCol

#热图可视化

pdf("heatmap.pdf", height=2.2, width=6)

pheatmap(data,

annotation=Type,

annotation_colors = ann_colors,

color = colorRampPalette(c("blue", "white", "red"))(50),

cluster_cols =F,

cluster_rows =F,

scale="row",

show_colnames=F,

fontsize=6,

fontsize_row=6,

fontsize_col=6)

dev.off()

**13.GSVA**

rm(list = ls())

#引用包

library(limma)

library(GSEABase)

library(GSVA)

library(pheatmap)

clusterFile="Cluster.txt" #分型输入文件

gmtFile="h.all.v7.5.1.symbols.gmt" #基因集文件

#读取表达输入文件,并对输入文件整理

load("merge_UC.RDATA")

rt=outTab1

exp=outTab1

dimnames=list(rownames(exp), colnames(exp))

data=matrix(as.numeric(as.matrix(exp)), nrow=nrow(exp), dimnames=dimnames)

data=avereps(data)

#GSVA分析

geneSets=getGmt(gmtFile, geneIdType=SymbolIdentifier())

gsvaResult=gsva(data,

geneSets,

min.sz=10,

max.sz=500,

verbose=TRUE,

parallel.sz=1)

gsvaOut=rbind(id=colnames(gsvaResult), gsvaResult)

write.table(gsvaOut, file="gsvaOut.txt", sep="\t", quote=F, col.names=F)

#读取cluster文件

cluster=read.table(clusterFile, header=T, sep="\t", check.names=F, row.names=1)

#数据合并

gsvaResult=t(gsvaResult)

sameSample=intersect(row.names(gsvaResult), row.names(cluster))

gsvaResult=gsvaResult[sameSample,,drop=F]

cluster=cluster[sameSample,,drop=F]

gsvaCluster=cbind(gsvaResult, cluster)

Project=gsub("(.*?)\\_.*", "\\1", rownames(gsvaCluster))

gsvaCluster=cbind(gsvaCluster, Project)

colnames(gsvaCluster)[which(colnames(gsvaCluster)=="cluster")]="cluster"

#差异分析

adj.P.Val.Filter=0.05

allType=as.vector(gsvaCluster$cluster)

comp=combn(levels(factor(allType)), 2)

for(i in 1:ncol(comp)){

#样品分组

treat=gsvaCluster[gsvaCluster$cluster==comp[2,i],]

con=gsvaCluster[gsvaCluster$cluster==comp[1,i],]

data=rbind(con, treat)

#差异分析

Type=as.vector(data$cluster)

ann=data[,c(ncol(data), (ncol(data)-1))]

ann[,1]="merged data"

data=t(data[,-c((ncol(data)-1), ncol(data))])

design=model.matrix(~0+factor(Type))

colnames(design)=levels(factor(Type))

fit=lmFit(data, design)

contrast=paste0(comp[2,i], "-", comp[1,i])

cont.matrix=makeContrasts(contrast, levels=design)

fit2=contrasts.fit(fit, cont.matrix)

fit2=eBayes(fit2)

#输出所有通路的差异情况

allDiff=topTable(fit2,adjust='fdr',number=200000)

allDiffOut=rbind(id=colnames(allDiff),allDiff)

write.table(allDiffOut, file=paste0(contrast, ".all.txt"), sep="\t", quote=F, col.names=F)

#输出显著的差异

diffSig=allDiff[with(allDiff, (abs(logFC)>0.1 & adj.P.Val < adj.P.Val.Filter )), ]

diffSigOut=rbind(id=colnames(diffSig),diffSig)

write.table(diffSigOut, file=paste0(contrast, ".diff.txt"), sep="\t", quote=F, col.names=F)

#聚类颜色

bioCol=c("#0066FF","#FF9900","#FF0000","#6E568C","#7CC767","#223D6C","#D20A13","#FFD121","#088247","#11AA4D")

ann_colors=list()

CluCol=bioCol[1:length(levels(factor(allType)))]

names(CluCol)=levels(factor(allType))

ann_colors[["cluster"]]=CluCol[c(comp[1,i], comp[2,i])]

#绘制差异通路热图

termNum=20

diffTermName=as.vector(rownames(diffSig))

diffLength=length(diffTermName)

if(diffLength<termNum){termNum=diffLength}

hmGene=diffTermName[1:termNum]

hmExp=data[hmGene,]

pdf(file=paste0(contrast,".heatmap.pdf"),height=6,width=10)

pheatmap(hmExp,

annotation=ann,

annotation_colors = ann_colors,

color = colorRampPalette(c("blue", "white", "red"))(50),

cluster_cols =F,

show_colnames = F,

gaps_col=as.vector(cumsum(table(Type))),

scale="row",

fontsize = 10,

fontsize_row=7,

fontsize_col=10)

dev.off()

}

1. **PCA**

rm(list = ls())

#引用包

library(limma)

library(ggplot2)

expFile="geneexpr.txt" #表达输入文件

clusterFile="Cluster.txt" #分型文件

#读取输入文件,并对输入文件进行整理

rt=read.table(expFile, header=T, sep="\t", check.names=F)

rt=as.matrix(rt)

exp=rt

dimnames=list(rownames(exp),colnames(exp))

data=matrix(as.numeric(as.matrix(exp)),nrow=nrow(exp),dimnames=dimnames)

data=avereps(data)

data=data[rowMeans(data)>0,]

data=t(data)

#PCA分析

data.pca=prcomp(data, scale. = TRUE)

pcaPredict=predict(data.pca)

write.table(pcaPredict, file="newTab.xls", quote=F, sep="\t")

#读取分型文件

cluster=read.table(clusterFile, header=T, sep="\t", check.names=F, row.names=1)

cluster=as.vector(cluster[,1])

#设置颜色

bioCol=c("#0066FF","#FF9900","#FF0000","#6E568C","#7CC767","#223D6C","#D20A13","#FFD121","#088247","#11AA4D")

CluCol=bioCol[1:length(levels(factor(cluster)))]

#可视化

PCA=data.frame(PC1=pcaPredict[,1], PC2=pcaPredict[,2], cluster=cluster)

PCA.mean=aggregate(PCA[,1:2], list(cluster=PCA$cluster), mean)

pdf(file="PCA.pdf", height=5, width=6.5)

ggplot(data = PCA, aes(PC1, PC2)) + geom_point(aes(color = cluster)) +

scale_colour_manual(name="cluster", values =CluCol)+

theme_bw()+

theme(plot.margin=unit(rep(1.5,4),'lines'))+

annotate("text",x=PCA.mean$PC1, y=PCA.mean$PC2, label=PCA.mean$cluster, cex=7)+

theme(panel.grid.major = element_blank(), panel.grid.minor = element_blank())

dev.off()
