# Supplementary material for: Identification of O-glycosylation related genes and subtypes in ulcerative colitis based on machine learning
Source: PLoS One. 2024 Dec 31;19(12):e0311495. doi: 10.1371/journal.pone.0311495 (PMC11687659; doi:10.1371/journal.pone.0311495)
Supplement: Attachment — Submitted filename: Response to Reviewers.pdf [file pone.0311495.s005.pdf]

Dear Editor and Reviewer:

Thank you for your decisions and constructive comments on our manuscript entitled "Identification of O-Glycosylation related genes and subtypes in Ulcerative Colitis based on machine learning". Those comments are all valuable and very helpful for revising and improving our paper. We have studied comments carefully and have made corrections which we hope meet with approval. The reviewers' comments are laid out below in italicized font and specific concerns have been numbered. Our response is given in normal font and changes/additions to the manuscript are given in the red text.

Responses to Reviewers comments:

Reviewer #1:

1. The quality of the writing is extremely poor and lacks scientific explanation. The choice of words for discussing any information requires more background studies and details technical descriptions. The abstract needed to be reframed and the introduction also required substantial corrections.

We apologize for the poor language of our manuscript. We have now worked on both language and readability, and really hope that the flow and language level have been substantially improved.

2. The background knowledge for the selection of the two different datasets for the study viz., GSE75214 and GSE92415 is not clear. The patient data from the selected population needed to be properly demonstrated. The authors are requested to follow https://doi.org/10.1371/journal.pone.0289064 for their reference. It was found that one of the datasets GSE92415 contained the data for UC patients treated with Golimumab, it is not mentioned anywhere in the manuscript and the rationale behind the selection of this particular study is not clear in the manuscript. It needed to be addressed properly.

We are sorry for our carelessness. In the methods section, we provide detailed background on the databases GSE75214 and GSE92415. In addition, the data for UC

patients treated with Golimumab were not included in our study (Line 77-87).

This study incorporated two datasets, GSE75214 (provided by the GPL6244 platform) and GSE92415 (provided by the GPL13158 platform). The GSE75214 database contains intestinal mucosal biopsies obtained endoscopically from UC patients (n=97) and healthy controls (n=11), and subsequently analyzed for gene expression by microarray. GSE92415 database enrolled 21 healthy subjects and 162 UC patients, including baseline before treatment (n=87) and post-treatment individuals (n=75), to evaluate the effect of golimumab (GLM) during induction treatment in moderately to severe UC. In this study, we selected only 87 untreated UC samples and 21 healthy control samples.

3. The selection of DEGs from the two discrete datasets is not clear. The authors should either first select the common DEGs between both the datasets and then screen for the Upregulated and downregulated genes or clearly describe their criteria for the selection process. The statistical significance and relevance of the screening and functional enrichment needed to be justified properly. Similar conditions apply to the LASSO analysis.

We apologize for the lack of clear instructions on the differential genes screening process. In order to increase the sample size, we included multiple datasets. However, there were batch effects among these datasets due to various factors, such as experimental time, batch, laboratory, and sample processing method. These batch effects were combined and eliminated to minimize technical differences and ensure data reliability and comparability. We combined GSE75214 and GSE92415 datasets and performed differential analysis using the R language limma package. According to the criteria of |logFC|>1 and adj.P. Val<0.05, 449 genes were up-regulated, and 233 genes were down-regulated.

4. The resolution of the figures needed to be modified along with the figure captions. The font and size of the text used in the figure are unclear and need to be modified.

We modified the captions of several figures to make them more accurate, like

Figure 4, and the resolutions are also adjusted appropriately. If any figure is not up to standard, please point it out and we will carefully modify it until it meets the PLOS ONE's standards. We sincerely thank the editor and all reviewers for the valuable feedback.

5. Regarding the establishment of the role of the critical genes in CRC/CAC, the authors may explore the differential expression of these critical genes for human CRC using the TCGA data. The authors can follow https://doi.org/10.1016/j.humgen.2023.201189 and https://doi.org/10.3389/fgene.2021.608313 for their reference.

We compared the expression of four hub genes in the TCGA and GEPIA databases, and found the differential expression is not exactly consistent with our study, as shown in the following pictures. For CRC studies, the expression levels of four key genes were also different in the two databases. In the TCGA database, the expression levels of four key genes in CRC were lower than those in the control group (Figure 1-4). Whereas, in the GEPIA database, MUC1, GXYLT2, and SEMA5A were highly expressed in CRC, thereinto SEMA5A had an obvious difference. ADAMTS1 was expressed at a low level in colorectal cancer (Figure 5-8). However, it is worth noting that the four core gene expression trends in subtype B were the same as those in previous research on UC, which we discussed amply in the Discussion section.

Colorectal cancer (CRC) and colitis-associated colorectal cancer (CAC) have crucial differences. CAC occurs due to complications of chronic inflammatory disorders of the colon, while most CRC arise from precancerous lesions or adenomatous polyps[1]. However, there are only CRC and no CAC data in TCGA, so we did not show the differential expression of these critical genes in the manuscript.

If the reviewers have better suggestions, we hope to strive for another opportunity to make modifications.

[1] Kasi A, Handa S, Bhatti S, Umar S, Bansal A, Sun W. Molecular Pathogenesis and Classification of Colorectal Carcinoma. Curr Colorectal Cancer Rep. 2020 Sep;16(5):97-106. doi: 10.1007/s11888-020-00458-z. Epub 2020 Aug 15. PMID:

32905465; PMCID: PMC7469945

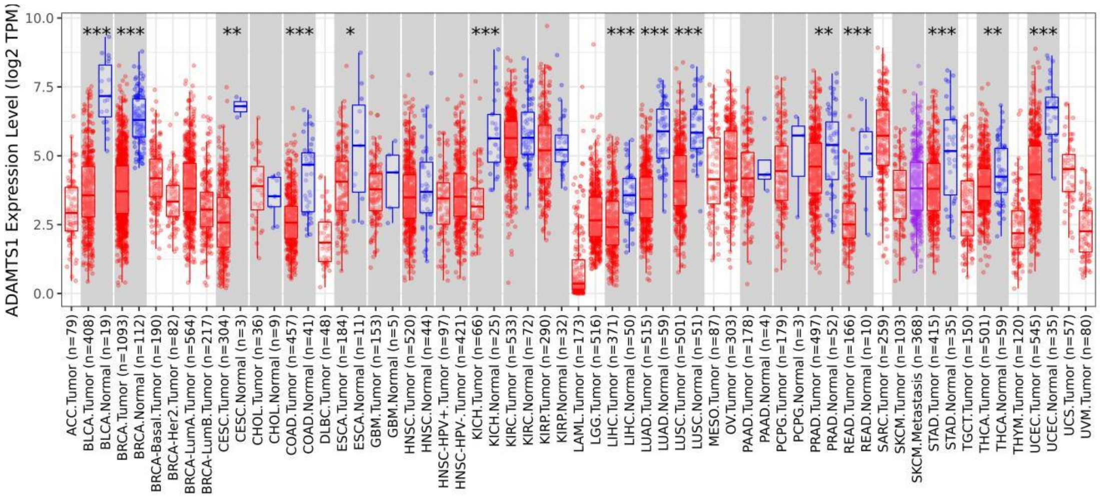

Figure 1. ADAMTS1 of TCGA

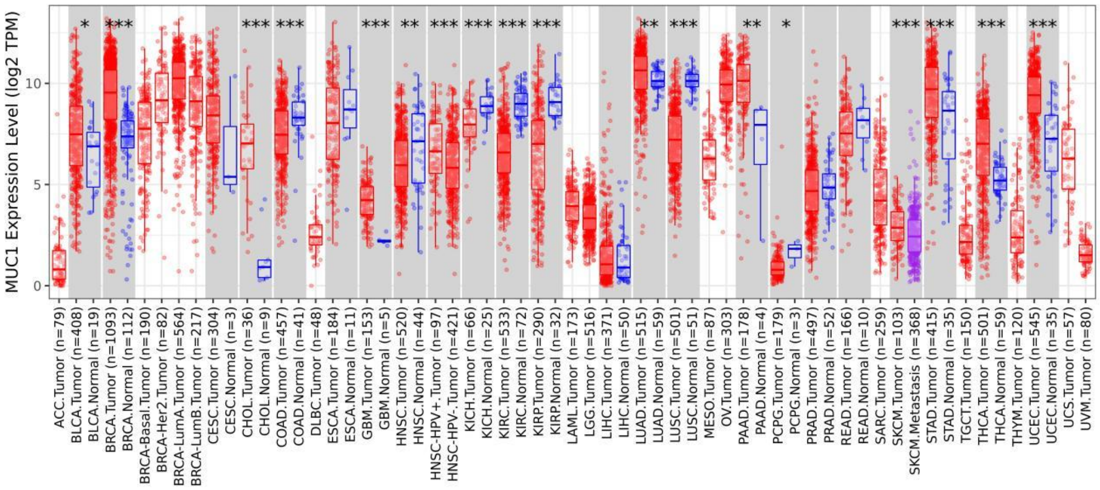

Figure 2. MUC1 of TCGA

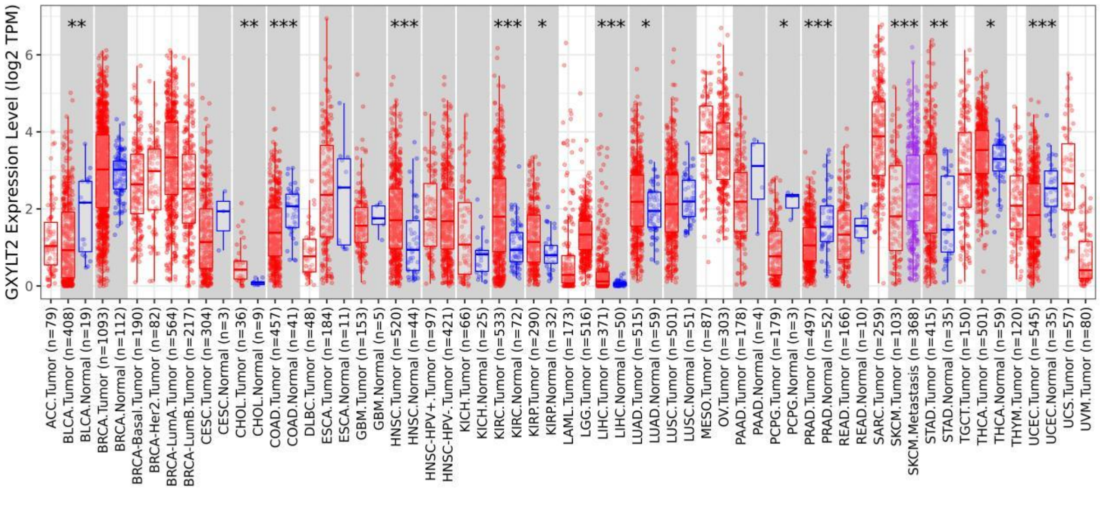

Figure 3. GXYLT2 of TCGA

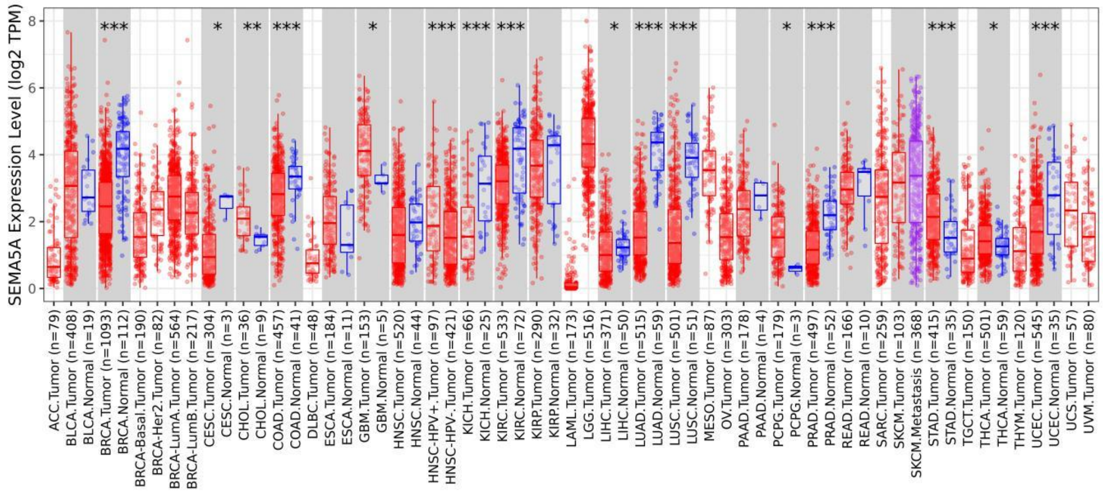

Figure 4. SEMA5A of TCGA

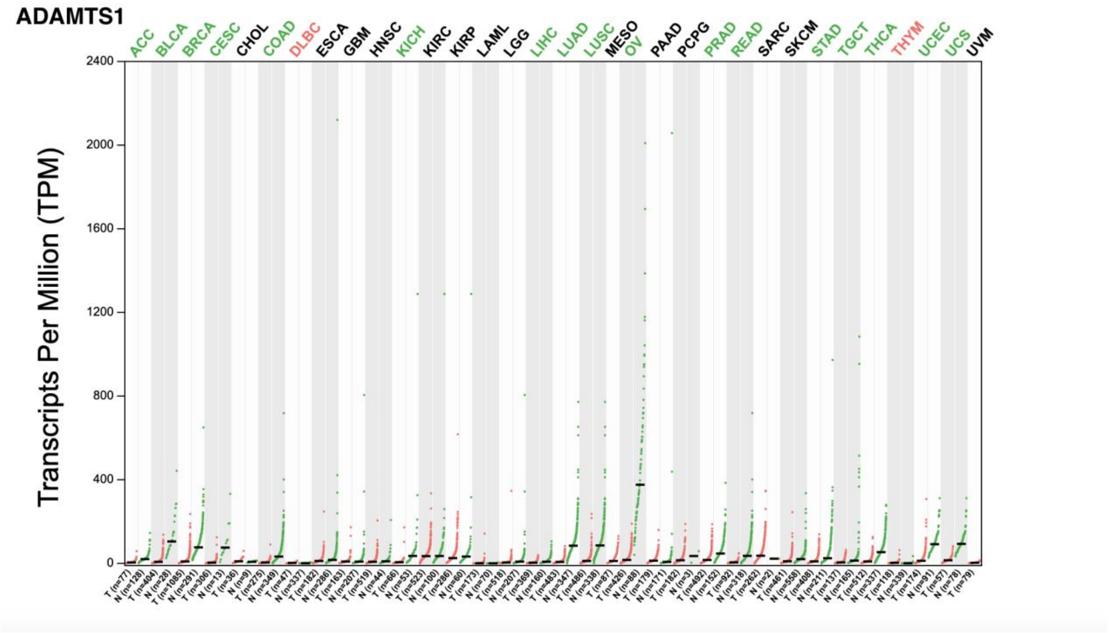

Figure 5. ADAMTS1 of GEPIA

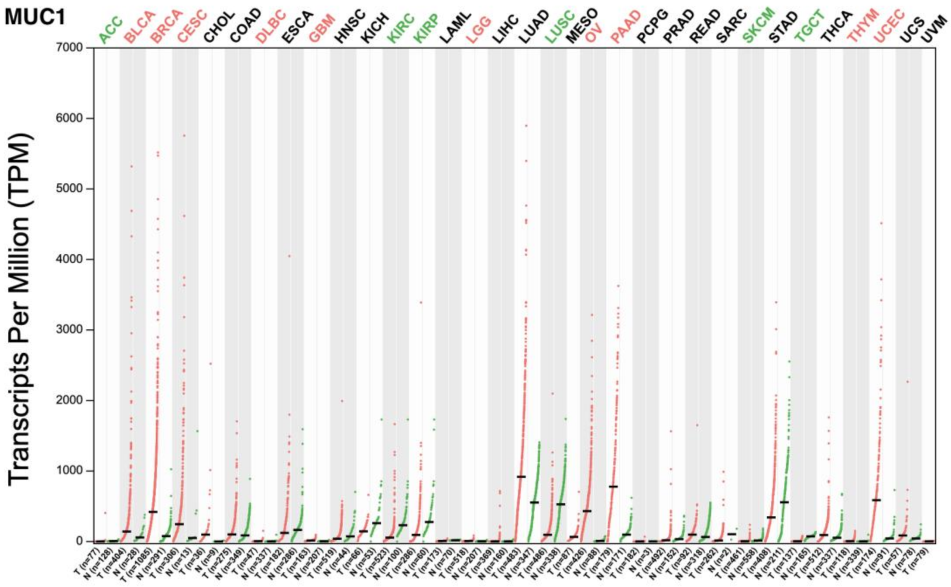

Figure 6. MUC1 of GEPIA

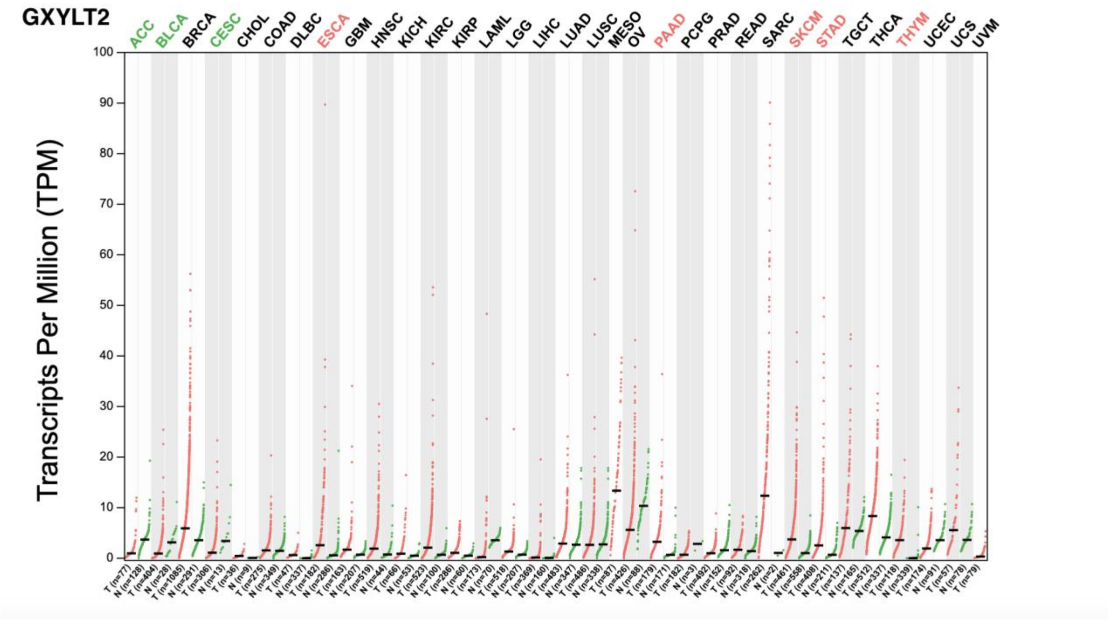

Figure 7. GXYLT2 of GEPIA

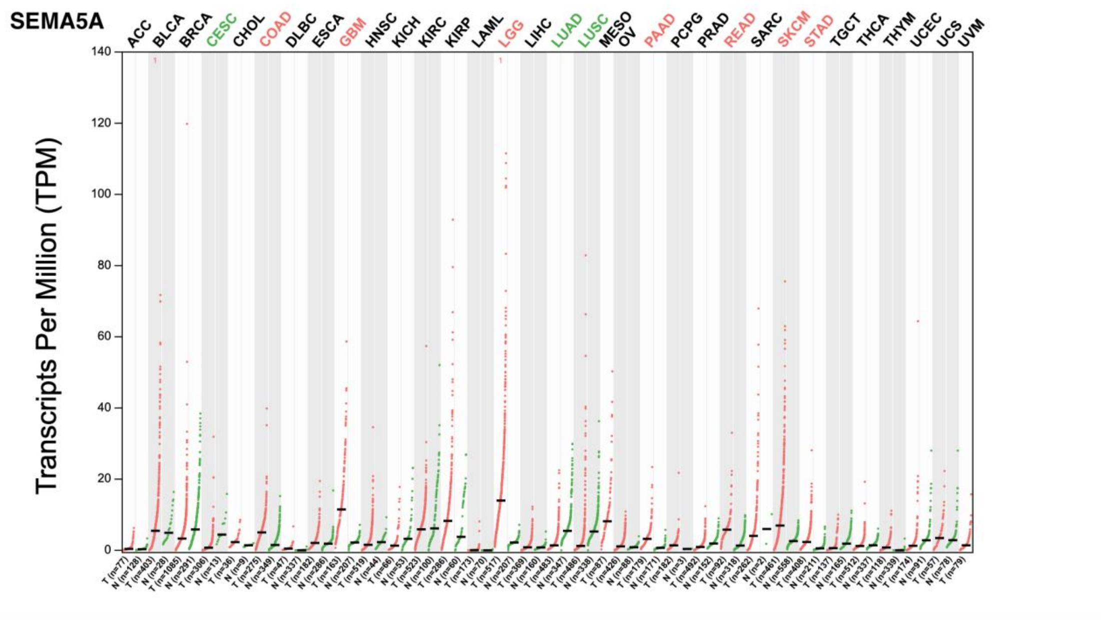

Figure 8. SEMA5A of GEPIA

Reviewer #2:

Thanks for your thoughtful review. In the results section, we do have a lot of shortcomings. We have made detailed revisions and added the details of bioinformatic analysis to make the logic more fluent and clearer.

1. Gene lists should be provided for differential expression analysis along with logFC values and p-values (e.g. for Figure 1 and 2).

Because the gene lists are too large to be displayed directly in the manuscript, they are uploaded as supplementary tables along with logFC values and p-values. In addition , we added the KEGG enrichment analysis tables of DEGs in the manuscript, as shown in Table 1 and 2.

2. For all figures, make sure to specify a legend for the scale used and any relevant cutoffs for fold change or p-value in the figure description (e.g for the heatmap in Figure 1). Furthermore, make sure to provide which figure you are referring to in the text (line 418).

We apologize for lack of rigor. In the revised manuscript, we have added the logFC

values and p-values, mainly Figure 1 and 9. Regarding the original 418 line section, we have reorganized the language and added the figure to make the description clearer (Line 97-100).

3. There is some ambiguity in the description of analysis (e.g. lines 427 to 429). For figure 4A-C, specify which tag genes were identified by each of the algorithms in the figures. The number of genes identified is inconsistent with the in-text description of the figure (lines 128 to 131). Furthermore, Figure 4E could be described in more detail in the text (specify positive/negative correlation or the exact correlation value).

In the section on methods and results of machine learning, we have provided a more detailed explanation (Line 128-135). For Figure 4A-C, the genes identified by each algorithm are listed in the revised manuscript. The explanation of Figures 4E and F has also been added (Line 307-319).

4. Relevant citations should be provided throughout the paper for all claims, (e.g. lines 25-30 and 43-45). Also provide references for packages and databases used (e.g. lines 152 and 427).

Based on your suggestions, we have checked the literature carefully and added more references on the definition of Glycosylation and the relationship between O-Glycosylation and diseases into the introduction part of the revised manuscript (Line 40-64). In Materials and methods part, we hope the complements of R packages references and websites of the databases involved can improve the credibility of the paper and meet the requirements of your journal. Besides, the key R packages have been uploaded as supporting information named "R packages".

5. The discussion of the results in the context of prior literature on UC would be of immediate interest for biologists and clinicians. In this regard, further development of the implications of the results can strengthen the paper.

At the beginning of the discussion, background information on ulcerative colitis was added (Line 510-521). The intestinal symptoms and complications of ulcerative

colitis can reduce the quality of life of patients, so it is necessary to explore its pathogenesis to guide treatment. We hope that the text added can arouse the interest of readers and look forward to further guidance from the reviewers.

Minor Issues:

1. The abstract and introduction mention the etiology of UC and the role of O-GlcNAcylation in various inflammatory diseases. However, the context from prior literature that the authors already provide could be strengthened by providing further details about which immune cells are known to play a role in promoting mucosal immune and inflammatory responses in UC.

According to the suggestions of the two reviewers, the abstract and introduction of this paper have been greatly improved. In the introduction and discussion sections, the correlation between immune cells and UC was supplemented (Line 31-35).

2. In the introduction, the authors mention that the level of protein O-GlcNAc changes (lines 32-35). Discuss these alterations and their implication further.

Thank you for the comment, the alterations and implications of O-GlcNAc changes are further described in lines 47-52.

3. In the results section, the article should introduce the two datasets before jumping into the analysis in the results section. Explicitly state what the differential expression analysis is comparing (e.g. UC vs. healthy controls in line 51) to better guide the reader.

We apologize for not providing a detailed introduction to the two datasets. Due to adjusting the order of methods and results, the background information of the datasets is presented in the methods section (Line 77-87), including methods and R packages for merging two datasets and screening for differentially expressed genes (Line 89-100).

4. Make sure the nomenclature for human genes/transcripts/proteins is correct

throughout the manuscript text and figures (e.g. in gene lists provided adjacent to heatmaps).

Thanks for your reminder, the nomenclature of human genes/transcripts/proteins in the text and figures are correct after checking.

5. Discuss the limitations of the study and suggest future experiments that can validate the findings.

In the original manuscript, the limitations were indeed a little brief. Now we have further discussed the limitations and added what experimental methods can be further supplemented in the future (Line 626-635).

6. Providing a table of the pathways along with p-values for the pathway enrichment analysis would be helpful.

The tables of the pathways along with p-values for the pathway enrichment analysis have been uploaded.

7. Some of the ambiguity in the analysis may be addressed by providing the code for replication by others.

We have provided the code by which the steps of this article can be repeated.

8. Language can be improved for clarity (e.g. lines 23-25). Please also proofread for typos (e.g. line 26 and 29).

We sincerely thank the reviewer for careful reading. In the resubmitted manuscript, the typo is revised, and the grammar is corrected.

We tried our best to improve the manuscript and made some changes, shown in detail in the file "Revised Manuscript with Track Changes". In addition, we would like to further confirm the authors' order of the paper "Identification of O-Glycosylation related genes and subtypes in Ulcerative Colitis based on machine learning". The following three authors, Yue Lu, Yi Su, and Nan Wang, contributed

equally to this work and be considered co-first authors. In addition, Dongyue Li was added as the 2nd set of equal contributors, whose e-mail is “lidongyue83@163.com”, and the author Shuyuan Zhang was deleted. We appreciate for editors and reviewers’ work earnestly, and hope correction will meet with approval. Once again, thank you very much for your comments and suggestions.

I appreciate your consideration. I am looking forward to hearing from you.

Sincerely,

Hongyu Xu

Institution and address: the First Affiliated Hospital of Harbin Medical University, 23 You Zheng Street, Nangang District, Harbin City, Heilongjiang Province, China

Telephone: 86-13903656899

E-mail: xuhongyu@ldy.edu.rs
