# Supplementary material for: Identification of O-glycosylation related genes and subtypes in ulcerative colitis based on machine learning
Source: PLoS One. 2024 Dec 31;19(12):e0311495. doi: 10.1371/journal.pone.0311495 (PMC11687659; doi:10.1371/journal.pone.0311495)
Supplement: Attachment — Submitted filename: Response to Reviewers.docx [file pone.0311495.s006.docx]

Dear Editor and Reviewer:

We thank the reviewer for the kind consideration and constructive comments on our manuscript. We have addressed your concerned in a point-by-point manner below, and hope that you will find the added information suitable and sufficient for publication. The reviewers’ comments are laid out below in italicized font and specific concerns have been numbered. Our response is given in normal font.

1. *Typos like "For visualization, the DEGs," (line 104) and numerous others should be corrected.*

Grammatically, our expression lacks rigor and we have made changes to make it more precise.(line104-105)

2. *Phrases like “involving three low expressions.” should be fixed to be informative and grammatically correct.*

Our representation is not accurate and concise enough. [Considering](https://www.baidu.com/s?rsv_idx=1&wd=considering%E7%BF%BB%E8%AF%91&fenlei=256&usm=2&ie=utf-8&rsv_pq=ca08931c003d6411&oq=%E9%89%B4%E4%BA%8E%E8%8B%B1%E8%AF%AD&rsv_t=c181eBBGaAq3dP6kdYRNGzPbNOFUVL6bQygYtFN0ig1QmgF9akII/J+dr6Q&sa=re_fy_huisou) Figure 3 has clearly shown the differential expression of genes between UC patients and controls, we deleted *“involving three low expressions.”*, which does not affect the reader's understanding.

3. *The font changes give a sloppy appearance in "Identification of differential expressed genes".*

In paragraph "Identification of differential expressed genes", the font is unified as “Times New Roman”, and the size is 14.

4. *Tables for GO analysis are not legible (exclude extraneous information) or shown as bar charts, with tables presented as supplementary information.*

According to the reviewer's suggestion, “qvalue” and “count” were deleted in the kegg tables to make the forms more concise.

We sincerely appreciate the time and effort invested by the reviewers in evaluating our manuscript. We are more than happy to make any further revisions to improve the paper and facilitate successful publication. Once again, thank you very much for your comments and suggestions.

Sincerely,

Hongyu Xu

Institution and address: the First Affiliated Hospital of Harbin Medical University, 23 You Zheng Street, Nangang District, Harbin City, Heilongjiang Province, China

Telephone: 86-13903656899

E-mail: xuhongyu@ldy.edu.rs
